# *Rrp1b*, a New Candidate Susceptibility Gene for Breast Cancer Progression and Metastasis

**DOI:** 10.1371/journal.pgen.0030214

**Published:** 2007-11-30

**Authors:** Nigel P. S Crawford, Xiaolan Qian, Argyrios Ziogas, Alex G Papageorge, Brenda J Boersma, Renard C Walker, Luanne Lukes, William L Rowe, Jinghui Zhang, Stefan Ambs, Douglas R Lowy, Hoda Anton-Culver, Kent W Hunter

**Affiliations:** 1 Laboratory of Population Genetics, National Cancer Institute, National Institutes of Health, Bethesda, Maryland, United States of America; 2 Laboratory of Cellular Oncology, National Cancer Institute, National Institutes of Health, Bethesda, Maryland, United States of America; 3 Laboratory of Human Carcinogenesis, National Cancer Institute, National Institutes of Health, Bethesda, Maryland, United States of America; 4 Epidemiology Division, Department of Medicine, University of California Irvine, Irvine, California, United States of America; Princeton University, United States of America

## Abstract

A novel candidate metastasis modifier, *ribosomal RNA processing 1 homolog B* (*Rrp1b*), was identified through two independent approaches. First, yeast two-hybrid, immunoprecipitation, and functional assays demonstrated a physical and functional interaction between Rrp1b and the previous identified metastasis modifier Sipa1. In parallel, using mouse and human metastasis gene expression data it was observed that extracellular matrix (ECM) genes are common components of metastasis predictive signatures, suggesting that ECM genes are either important markers or causal factors in metastasis. To investigate the relationship between ECM genes and poor prognosis in breast cancer, expression quantitative trait locus analysis of polyoma middle-T transgene-induced mammary tumor was performed. ECM gene expression was found to be consistently associated with *Rrp1b* expression. In vitro expression of *Rrp1b* significantly altered ECM gene expression, tumor growth, and dissemination in metastasis assays. Furthermore, a gene signature induced by ectopic expression of *Rrp1b* in tumor cells predicted survival in a human breast cancer gene expression dataset. Finally, constitutional polymorphism within *RRP1B* was found to be significantly associated with tumor progression in two independent breast cancer cohorts. These data suggest that *RRP1B* may be a novel susceptibility gene for breast cancer progression and metastasis.

## Author Summary

Metastasis, which is defined as the spread of malignant tumor cells from their original site to other parts of the body, accounts for the vast majority of solid cancer-related mortality. Our laboratory has previously shown that host germline-encoded variation modifies primary tumor metastatic capacity. Here, we detail how germline-encoded *Rrp1b* variation likely modulates metastasis. In mice, constitutional *Rrp1b* variation correlates with ECM gene expression, which are genes commonly differentially regulated in metastasis prone tumors. Furthermore, we demonstrate that *Rrp1b* expression levels are modulated by germline variation in mice with differing metastatic propensities, and that variation of *Rrp1b* expression in a highly metastatic mouse mammary tumor cell line modifies progression. Differential *RRP1B* functionality also appears to play an important role in human breast cancer progression. Specifically, we demonstrate that a microarray gene expression signature indicative of differential *RRP1B* expression predicts breast cancer-specific survival. Furthermore, we show that germline-encoded *RRP1B* variation is associated with markers of outcome in two breast cancer populations. In summary, these data suggest that *Rrp1b* may be a germline-encoded metastasis modifier in both mice and humans, which leads to the possibility that knowledge of *RRP1B* functionality and variation in breast cancer might facilitate improved assessment of prognosis.

## Introduction

Most cancer-related mortality is a consequence of metastasis, and the vast majority of deaths from breast cancer, the most common malignancy of women in the United States [1], are attributable to disseminated disease. Disseminated breast cancer is still considered incurable in spite of therapeutic advances [2], and a more comprehensive understanding of the biology of tumor progression is therefore necessary to facilitate development of improved treatments. This includes the ability to spare women at low risk of metastasis from needless additional therapy, while allowing earlier initiation of aggressive treatment to reduce the incidence and extent of metastasis in women with poorer prognoses.

We previously demonstrated the significant influence of germline variation on tumor progression [3,4], which allowed us to identify the first known heritable mouse gene that modulates metastasis [5,6], the Rap-GTPase activating protein (GAP) *Sipa1* [7]. Subsequent human studies demonstrated that *SIPA1* polymorphisms are associated with metastatic cancer [7] and poor outcome in breast cancer [8], validating the utility of the highly metastatic polyoma middle-T (PyMT) transgenic mouse model to identify relevant human metastasis modifiers. The current study represents the convergence of two parallel strategies to enhance our understanding of the role of heritable factors in metastasis. Using in vitro, genetic, and epidemiologic analyses, we have identified ribosomal RNA processing 1 homolog B (Rrp1b) as a factor that physically interacts with the metastasis modifier gene, *Sipa1*, modulates elements of metastasis predictive gene expression signatures, suppresses tumor progression in animal models, and is associated with progression and survival in pilot human breast cancer epidemiology cohorts. This integrated approach suggests that *Rrp1b* is a novel tumor progression and metastasis susceptibility locus in both mice and humans.

## Results

### Rrp1b Forms a Complex with Sipa1 and Inhibits Sipa1 Gap Activity

Previous mouse studies demonstrated that a polymorphism in *Sipa1* in the region encoding a PDZ protein–protein interaction domain is associated with metastasis [7]. Yeast two-hybrid screening of Sipa1 was therefore performed to identify additional genes potentially involved in metastasis (Table S1). Following sequence alignment, 29 clones were found to bind to at least one of the SIPA1 baits (Table S2). One of these was RRP1B (the human homolog of Rrp1b), which was identified by a probe spanning the PDZ domain.

**Figure 1:**
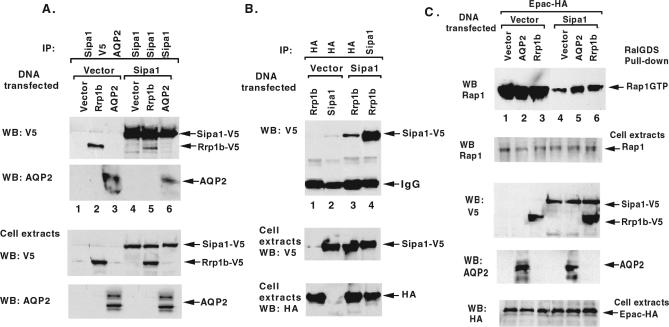
Rrp1b Forms a Complex with Sipa-1 and Inhibits its RapGAP Activity (A) and (B) Complex formation between Rrp1b and Sipa1, and between AQP2 and Sipa1. AQP2 or Rrp1b-V5 (A) or Rrp1b-HA (B) was expressed singly or coexpressed with Sipa1-V5 in 293 cells as indicated. In (A), anti-Sipa1 immunocomplexes were immunoblotted with anti-V5 antibodies (top western blot (WB) panel) or anti-AQP2 antibodies (next WB panel). In (B), anti-Rrp1b-HA immunocomplexes were immunoblotted with anti-Sipa1-V5 antibody (WB panel). In the two lower panels of A and B, the immunoblots show the expression in cell extracts of exogenous Rrp1b-V5, Sipa1-V5, AQP2, and Rrp1b-HA. (C) Inhibition of Sipa1 RapGAP Activity by AQP2 and Rrp1b. In cells expressing Epac, Sipa1-V5 was expressed with AQP2 or Rrp1b-V5 as indicated. The in vivo Rap1GTP levels were assayed by RalGDS-RBD pull-down (top panel). In the lower panels, the immunoblots show the expression of endogenous Rap1, and the exogenous proteins: Sipa1-V5, Rrp1b-V5, AQP2, and Epac-HA. The Rap1 and EpacHA immunoblots also serve as loading controls.

To confirm the interaction, HEK293 cells were cotransfected with epitope-tagged mouse Rrp1b and Sipa1. AQP2, which also interacts with the PDZ domain of Sipa1, was cotransfected with Sipa1 as a positive control. Cell extracts were then immunoprecipitated with Sipa1 antibodies and blotted with V5-antibodies (V5 was the epitope fused to Rrp1b in this experiment), revealing an Rrp1b-specific band (Figure 1A, upper panel, lane 5). Conversely, when HA-tagged Rrp1b was cotransfected with V5-tagged Sipa1, immunoprecipitation with an HA-antibody followed by western blotting yielded a Sipa1-specific band (Figure 1B, upper panel, lane 3).

As further validation, the functional consequence of the Rrp1b–Sipa1 interaction on the Rap-GTPase enzymatic activity of Sipa1 was examined. HEK293 cells were cotransfected with a Rap exchange factor, Epac, and Sipa1 in the presence of AQP2 or Rrp1b (Figure 1C). AQP2, which has been shown previously to interfere with the RapGAP activity of Sipa1 [7], was used as a positive control. In the absence of Sipa1, Epac induced an increase in Rap-GTP, regardless of whether the cells also expressed AQP2 or Rrp1b (upper panel, lanes 1–3), indicating that Rrp1b did not directly affect Rap-GTP levels. As expected, the presence of Sipa1 reduced Epac-induced Rap-GTP levels (upper panel, lane 4). This reduction was partially inhibited by AQP2 or Rrp1b (upper panel, lanes 5 and 6, respectively). Thus Rrp1b, like AQP2, inhibits the RapGAP activity of Sipa1.

### Expression QTL Mapping in AKXD Recombinant Inbred Mice

Examination of published reports describing primary human breast tumor expression profiles predicting metastasis or disease outcome reveals a common association with the expression levels of extracellular matrix (ECM) genes [9–11]. Similar ECM-rich metastasis-predictive signatures also exist in PyMT-induced mouse mammary tumors [12]. The consistent association of ECM gene expression levels with outcome suggests that differential ECM gene expression is either a causative factor or marker of metastatic potential.

**Figure 2:**
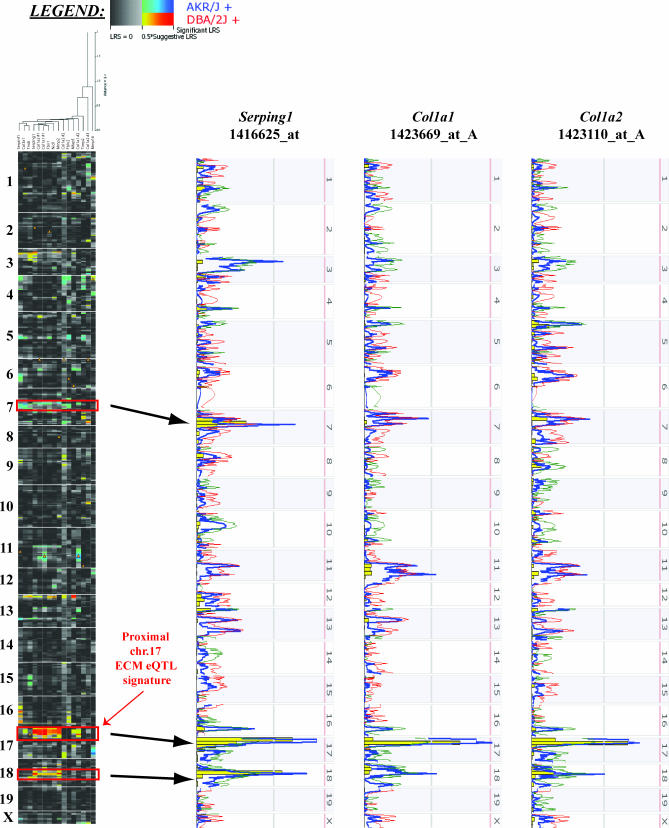
eQTL Analysis in the AKXD RI Mouse Panel Analysis of metastasis class-predictive ECM gene component expression patterns in PyMT transgene-induced tumors in AKXD recombinant inbred mice reveals the presence of three relatively consistent eQTLs on chromosomes 7, 17, and 18. The diagram on the left shows an eQTL cluster map generated with WebQTL. The warm hues reflect an LRS for increased transcription in mice with the DBA/2J genotype at any given locus, and the cool hues represent LRS for elevated transcription in mice with the AKR/J allele. The strongest degree of linkage is observed with the chromosome 17 eQTL, as illustrated in the three QTL scans on the right. In total, nine of the 16 metastasis-predictive probes displayed a LRS that was at least suggestive of linkage (denoted by the warm hues on the heat map to the left) suggesting that their expression is controlled in some respect by the chromosome 17 locus. These probes are located within the metastasis-predictive ECM genes *Serping1*, *Nid1*, *Col1a1*, *Col1a2*, *Col3a1*, *Fbn1*, and *Mmp2*. Rrp1b lies in very close physical proximity to the peak LRS within the chromosome 17 locus and displays a high degree of correlation of expression with a number of class-predictive ECM genes.

In a set of experiments performed concurrently with our efforts to identify proteins interacting with SIPA1, the relationship between ECM gene expression and metastasis susceptibility was further characterized by investigating the inherited origins of metastasis-predictive gene signatures. Specifically, expression quantitative trait locus (eQTL) mapping of ECM gene expression was performed to identify genomic regions associated with ECM gene expression (see Text S1). To achieve this, we analyzed microarray data derived from PyMT-induced primary tumors in the AKXD RI panel recombinant inbred (RI) mice [13,14], a panel of RI mice derived from high metastatic potential AKR/J and low metastatic potential DBA/2J strains [3]. An Internet-based analytical package/repository that allows for analysis of RI microarray expression data called WebQTL [15,16] was used to map ECM eQTLs. Reproducible, statistically significant or suggestive ECM eQTLs were discovered on chromosomes 7, 17, and 18 (see Figure 2 and Text S1), implying that loci from these three regions regulate much of the metastasis-predictive ECM gene expression in AKXD tumors. The chromosome 17 locus (Figure S1) was of particular interest, as its peak linkage region (∼29.5 Mb) colocalizes with a previously described metastasis efficiency and tumor growth kinetics QTL [6], and encompasses the physical location of *Rrp1b* (∼29.9 Mb).

Based on the current prevailing hypothesis that most modifiers are likely to result from modest variations in gene expression levels or mRNA stability [16,17], identification of potential candidates for the chromosome 17 ECM modifiers was performed by correlation analysis. Genes were correlated with ECM gene expression on a genome-wide scale using the Trait Correlation function of WebQTL. The results were subsequently filtered to examine only those genes that were present under each of the ECM eQTL peaks. Thirty genes located within a genomic region spanning the peak likelihood ratio statistic (LRS) score (physical locations on chromosome 17 ∼ 18–40 Mb) displayed both a high degree of correlation and a low *p* value with regard to expression of two or more of the nine probes within metastasis-predictive ECM genes (Table S3). *Rrp1b* was one of those genes that displayed high levels of expression correlation with ECM gene probes (Table S3). This gene was selected for further analysis for the following reasons: (a) its physical proximity to the peak eQTL linkage; (b) its apparent correlation with expression of various metastasis-predictive ECM genes; and (c) that it had also been identified as interacting with the metastasis efficiency modifier *Sipa1*. The effects of other genes within the eQTL linkage region upon metastasis remain under investigation, but their potential role in the modulation of metastasis efficiency (if any) is beyond the scope of the current study.

### Ectopic Expression of *Rrp1b* Modulates ECM Gene Expression

To confirm the role of *Rrp1b* in the regulation of ECM expression, cell lines stably over-expressing *Rrp1b* were generated in the highly metastatic mouse mammary tumor cell lines Mvt-1, which is derived from FVB/NJ mice [18] and 4T1, which is derived from BALB mice [19]. Multiple individual clones were generated by clonal dilution, and ectopic expression of *Rrp1b* confirmed by quantitative real-time PCR (qPCR) (ratio of *Rrp1b* expression in Mvt-1/*Rrp1b* versus controls = 3.28 ± 0.41; *p* = 0.001 and ratio of *Rrp1b* expression in 4T1/*Rrp1b* versus controls = 4.55 ± 0.95; *p* = 0.015). Metastasis-predictive ECM gene expression was then quantified, and expression of eight of the 12 quantified ECM genes (*Col1a1*, *Col3a1*, *Col6a2*, *Fbln2*, *Fbn1*, *Mfap5*, *Serpinf1*, and *Serping1*; see Table 1) was significantly changed in response to ectopic *Rrp1b* expression in the Mvt-1 cell line. Expression analysis of five of six significantly dysregulated genes in the 4T1 cell lines changed in the same direction as the Mvt-1/*Rrp1b* cells (Table 1), suggesting that *Rrp1b* modulation of ECM genes was not a unique characteristic of the Mvt-1 epithelial cell line. To confirm that these results were not an artifact of the Mvt-1 and 4T1 tumor cell lines, the experiment was repeated in NIH-3T3 fibroblasts. Of the six metastasis-predictive ECM genes dysregulated in all three cell lines, four showed the same profile in response to ectopic *Rrp1b* expression. The remaining discrepancies are likely due to experimental variability, differently regulated genes in epithelial (i.e., Mvt-1, 4T1) and mesenchymal cells (i.e., NIH-3T3), or the effects of the differing genetic backgrounds of the three cell lines.

**Table 1:**
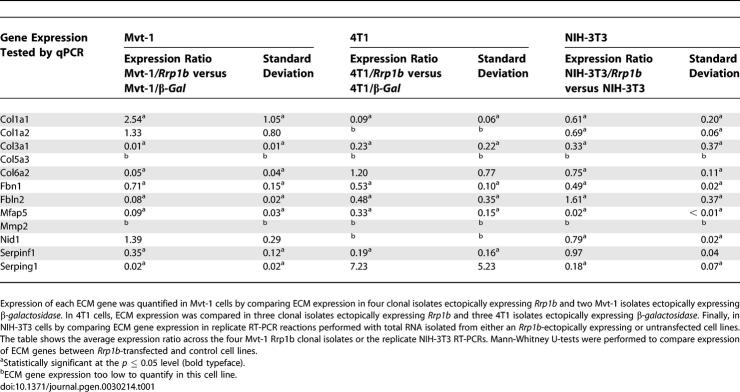
Ectopic Expression of *Rrp1b* in the Highly Metastatic Mouse Mammary Tumor Cell Lines Mvt-1 and 4T1, and NIH-3T3 Mouse Fibroblasts Modulates Expression of Various Metastasis Predictive ECM Genes

Growth curves were plotted for the Mvt-1 cell lines to confirm that the observed changes in gene expression in did not result from differential cellular growth rates (Figure S2). The growth of *Rrp1b*-transfected cell lines did not significantly differ from the growth rate of the control cell lines implying that the observed differences in metastasis-predictive gene expression are intrinsically related to the effects of *Rrp1b* rather than secondary to reduced growth kinetics. Similarly, no differences in growth rates were observed with the 4T1 and NIH-3T3 cells ectopically expressing *Rrp1b* compared to control cell lines (unpublished data).

### Tumor Growth and Metastatic Potential Are Reduced by Ectopic Expression of *Rrp1b*

**Figure 3:**
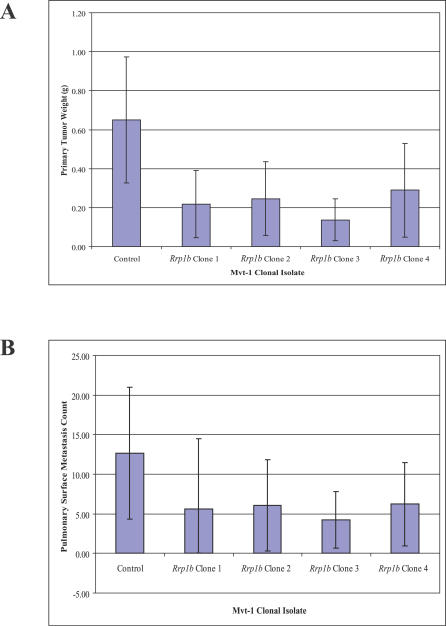
Ectopic Expression of *Rrp1b* Reduces (A) Tumor Growth and (B) Metastasis Burden in the Highly Metastatic Mvt-1 Cell Line Mvt-1 cell lines were stably transfected with a mammalian expression vector encoding either *Rrp1b* or *β-galactosidase*. Following isolation of individual clones by serial dilution and confirmation of ectopic gene expression, clonal isolates were subcutaneously implanted in FVB/NJ mice. Those mice implanted with Mvt-1 clones ectopically expressing *Rrp1b* developed smaller primary tumors and fewer pulmonary metastases compared to mice implanted with a clonal isolate ectopically expressing *β-galactosidase*.

Spontaneous metastasis assays were performed by subcutaneously implanting equal amounts of either Mvt-1/*Rrp1b* or Mvt-1/*β-galactosidase* clones into virgin FVB/NJ female mice. Specifically, the in vivo growth characteristics of four Mvt-1/*Rrp1b* clones were compared to that of one Mvt-1/*β-galactosidase* cell line. Since previous experiments have demonstrated that the in vitro and in vivo growth characteristics of the multiple independent isolates of the control cell line are virtually identical to those of the wild-type cell line, only one such cell line was used to minimize the number of animals in accordance with National Cancer Institute Animal Care and Use guidelines. Tumor weight and lung surface metastasis count were quantified following a four-week incubation period. Both tumor growth and lung surface metastasis were significantly reduced in Mvt-1/*Rrp1b* clonal isolates. Average tumor weight was 240 mg ± 200 mg for the *Rrp1b* clones compared to 600 mg ± 270 mg for the *β-galactosidase* clone (*p* < 0.001) (Figure 3A), and average lung surface metastasis count being 5.7 ± 6.0 for the *Rrp1b* clones compared to 12.6 ± 8.4 for the *β-galactosidase* clone (*p* = 0.010) (Figure 3B).

### *Rrp1b* Promoter Is More Active in Low-Metastatic DBA/2J Than in High-Metastatic AKR/J

Genomic sequencing of *Rrp1b* was performed from high (AKR/J, FVB/NJ) and low (I/lnJ, DBA/2J, and NZB/B1NJ) metastatic inbred strains to identify polymorphisms that might account for the differential ECM gene expression. Of particular interest were polymorphisms identified in the AKR/J and DBA/2J strains, since these are the progenitors of the AKXD RI panel. In addition to multiple intronic polymorphisms, the AKR/J *Rrp1b* proximal promoter contained two adenosine insertion polymorphisms located 1,132 bp and 1,540 bp upstream of the transcription initiation site. *Rrp1b* polymorphisms in all strains are listed in Table S4.

To examine the functional consequences of the AKR/J promoter polymorphisms, a region 1.67 kb upstream of the transcription initiation site of *Rrp1b* from AKR/J and DBA/2J was cloned into pBlue-TOPO (Invitrogen). Following normalization for transfection efficiency it was found that the AKR/J proximal promoter activity was reduced 30% relative to its DBA/2J counterpart (*p* < 0.001) (Figure S3; Table S5) implying a subtle functional difference in *Rrp1b* functionality between the high and low metastatic genotypes.

Given the outcome of the promoter activity experiments, we would expect to observe differential expression of *Rrp1b* in tissue derived from the AKXD ancestral AKR/J and DBA/2J strains. To test this hypothesis, we quantified expression of *Rrp1b* in normal mammary tissue from AKR/J or DBA/2J genotype mice. Following total RNA extraction from the mammary tissue from three individual AKR/J mice and three DBA/2J mice, reverse-transcription PCR (RT-PCR) was used to synthesize cDNA, and *Rrp1b* expression determined using qPCR. *Rrp1b* expression was found to be ∼30% less in normal mammary tissue derived from high metastatic potential AKR/J mice compared to mammary tissue from the low metastatic potential DBA/2J genotype (AKR/J normalized relative *Rrp1b* quantity = 1.21 ± 0.20, DBA/2J relative expression = 1.70 ± 0.30; Mann Whitney U-test *p* = 0.0495; Table S6). Combined, the in vitro and in vivo data imply that germline polymorphism, in the form of proximal promoter polymorphism, is causing differential functionality of *Rrp1b* in genetic backgrounds of differential metastatic capacity.

### A Gene Expression Signature Indicative of Ectopic Expression of *Rrp1b* Predicts Survival in Human Breast Cancer

If *RRP1B* is at least partially responsible for the presence of the ECM components of metastasis predictive gene signatures, it would suggest that a signature of *RRP1B* activation or expression [20] might also be predictive of breast cancer survival. To test this hypothesis, Affymetrix microarrays were used to compare gene expression in four Mvt-1/*Rrp1b* clonal isolates and three Mvt-1/*β-galactosidase* clonal isolates. An *Rrp1b* expression signature was identified using the Class Comparison tool of BRB ArrayTools was performed, using a two-sample t-test with random variance univariate test. *p*-Values for significance were computed based on 10,000 random permutations, at a nominal significance level of each univariate test of 0.0001. A total of 1,739 probe sets representing 1,346 genes passed these conditions. Significantly upregulated and downregulated probes according to these criteria are listed in Tables S7 & S8, respectively.

**Figure 4:**
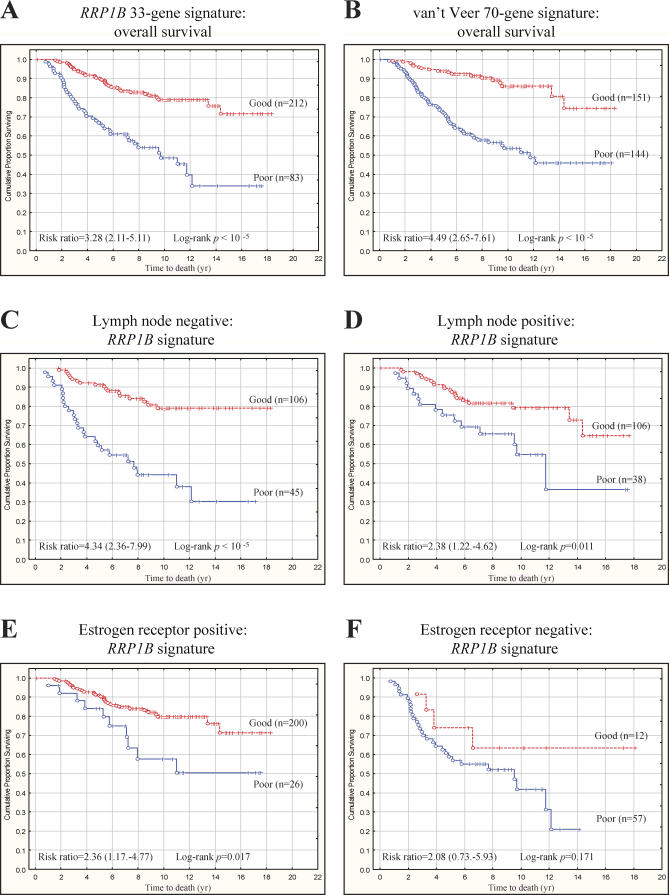
The *RRP1B* Microarray Expression Signature Predicts Survival into Good and Poor Outcome Groups Based on Tumor Gene Expression (A) The *RRP1B* signature was highly predictive of overall survival in the Dutch Rosetta dataset, with the cumulative proportion survival being estimated to be 72% versus 34% for the good and poor prognosis *RRP1B* signatures, respectively (*RRP1B* signature HR = 3.28, 95% CI = 2.11–5.11). (B) Indeed, it appears that the *RRP1B* signature possesses a similar ability to predict survival in this dataset than the 70-gene signature described by van't Veer et al. [10]. Specifically, the survival for the good and poor prognosis 70-gene signatures were estimated to be 73% versus 47%, respectively (70 gene signature HR = 4.49, 95% CI = 2.65–7.61). (C) To stratify patients more adequately based upon their disease characteristics, *RRP1B* signature gene expression was examined with respect to tumor LN and ER status. Overall survival in the Dutch Rosetta patients with negative LNs was 79% versus 30% for the good and poor prognosis *RRP1B* signatures, respectively (HR = 4.34, 95% CI = 2.36–7.99). (D) Similarly, the *RRP1B* signature could sub-stratify patients with positive LNs into good and poor prognosis groups, with survivals being 65% versus 37%, respectively (HR = 2.38, 95% CI = 1.22–4.62). (E) A similar stratification effect by tumor *RRP1B* signature gene expression was observed in patients with ER positive tumors, with an overall survival in patients with ER positive tumors being 72% versus 50% for the good and poor prognosis *RRP1B* signatures, respectively (HR = 2.36, 95% CI = 1.17–4.77). (F) However, it did not prove possible to stratify patients with ER negative tumors based upon their *RRP1B* expression signature. This likely reflects the lack of individuals with this disease subtype in this cohort.

A human *RRP1B* gene expression signature was generated by mapping the differentially regulated genes from mouse array data to human Rosetta probe set annotations [10]. One hundred ninety six genes from the mouse data could be mapped to the available Rosetta Hu25K chip annotations. The 295 samples of the Rosetta data set [10] were clustered into one of two groups representing high and low levels of *RRP1B* activation in primary tumor samples in an unsupervised manner based on the 196 significantly differentially expressed *RRP1B* signature genes on the Hu25K chip. Kaplan-Meier survival analysis was performed to investigate whether there was a survival difference between groups. A significant survival difference was observed implying that the level of activation of *RRP1B* or *RRP1B*-associated pathways within a tumor, presumably because of either somatic mutation or germline polymorphism, may be an important determinant of the overall likelihood of relapse and/or survival (Figure 4A). Further analysis indicated that survival was associated primarily because of the effects of 33 genes (Table S9). The degree of survival difference represented by the 33-gene *RRP1B*-induced gene expression signature was similar to the original 70-gene signature described by van't Veer and colleagues [10] (Figure 4B).

Patient samples were stratified by estrogen receptor (ER) and lymph node (LN) status, two clinically relevant prognostic markers, to determine whether the *RRP1B* signature might provide additional clinical stratification. Expression of the *RRP1B* signature in bulk primary tumor tissue predicted outcome in patients that were both LN negative and LN positive and patients with ER positive tumors (Figure 4C, 4D & 4E, respectively). Patients with ER negative tumors did not show a significant survival benefit (Figure 4F). However, this may be due to the limited sample size and needs to be clarified with additional studies.

### A Polymorphism in Human *RRP1B* Is Associated with Improved Outcome: Orange County Cohort

To validate a possible role of *RRP1B* in human cancer, a case-only pilot breast cancer association study was performed to assess the role of a nonsynonymous SNP within the human homolog of *Rrp1b* (dbSNP ID: rs9306160; 1421G→A, Pro436Leu) in human disease (Table S10). The variant A allele frequency in this Caucasian cohort was 0.362 (*n* = 269). Univariate analysis revealed a significant difference with respect to disease stage (localized versus nonlocalized *p* = 0.006; Table 2). Of the 130 patients with localized disease at diagnosis, 85 (65%) were carriers of the variant allele compared with 74 of 139 (53%) of those with advanced regional or metastatic disease. Significant associations were also observed with tumor ER and progesterone receptor (PR) status, the presence of LN disease, and primary tumor grade. The variant allele was more frequent among patients with ER positive and PR positive primary tumors: 122 of 190 individuals (64%) with ER positive tumors had the variant allele versus 25 of 54 patients (46%) with ER negative tumors (*p* = 0.001), and 104 of 160 individuals (65%) with PR positive tumors versus 41 of 82 subjects (50%) of those with PR negative tumors (*p* = 0.001). Furthermore, the AG and AA genotypes were more frequent among patients with well to moderately differentiated tumors (76 of 116 individuals (66%) versus 49 of 96 subjects (51%) with poorly differentiated tumors; *p* = 0.001). The variant allele was also more frequent among LN negative patients when compared with LN positive patients (81 of 125 patients (65%) with no positive LN versus 65 of 123 individuals (53%) with ≥1 LN; *p* = 0.033). No significant differences were observed with respect to primary tumor size and variant allele status did not influence disease-free survival in this cohort. Multivariate analysis that included age at diagnosis as covariate confirmed these results (Table 2), which again demonstrated that the variant allele was associated with a number of indicators of improved outcome.

**Table 2:**
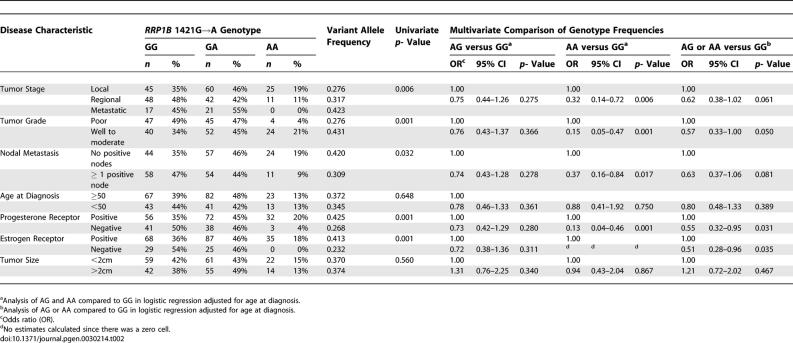
Genotype and Allele Frequency of *RRP1B* 1421G→A SNP in the Orange County Cohort by Tumor Characteristic: Univariate and Multivariate Analyses

### Analysis in a Breast Cancer Cohort from the Greater Baltimore Area

**Figure 5:**
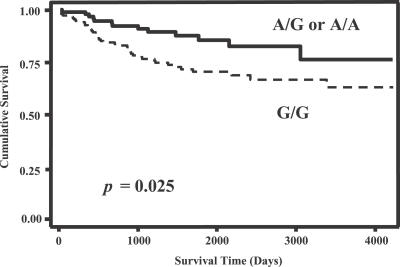
*RRP1B* 1421G→A SNP and Breast Cancer Survival Kaplan-Meier survival analysis. The survival of patients with the variant allele (A/G or A/A; *n* = 92) is significantly better than the survival of patients who are homozygous carriers of the common allele (G/G; *n* = 150). Two-sided log-rank test: *p* = 0.025.

*RRP1B* SNP analysis was performed in a second small pilot cohort consisting of 248 surgical breast cancer patients (58% African-American, 42% Caucasian) from the greater Baltimore area (Table S11) to attempt to replicate the findings of the initial cohort study. Stratification on race/ethnicity and age at disease onset in the Baltimore cohort indicated that neither of these variables was a significant confounding factor. Consistent with the Orange County cohort, the variant A allele was less frequent in patients with a high stage or poor grade tumor, with ER negative or PR negative tumors, and with a LN positive disease (Table 3). Most associations between the A allele and tumor markers were best explained by assuming an additive effect of the variant allele (Tables 2 & 3), however, studies in larger populations are required to better define the relative effect of the variant allele on outcome markers in breast cancer. We also examined the association between the 1421G→A SNP and breast cancer survival by assuming a dominant effect of the variant allele on survival, a model that best reflects our survival data. Carriers of the variant allele had a significantly better breast cancer-specific survival compared to homozygous carriers of the common allele (Figure 5). Multivariate Cox regression analysis with adjustments for age at diagnosis, race, ER status, tumor–node–metastasis (TNM) stage, and chemotherapy, further confirmed this observation. Patients who carried the variant allele had improved survival when compared to patients with the G/G genotype (hazard ratio of death (HR) = 0.46; 95% confidence interval (CI) = 0.21–0.97). This effect was stronger among patients with an ER positive tumor (HR = 0.17; 95% CI = 0.04–0.70) suggesting that *RRP1B* may have a particular function in the ER pathway.

**Table 3:**
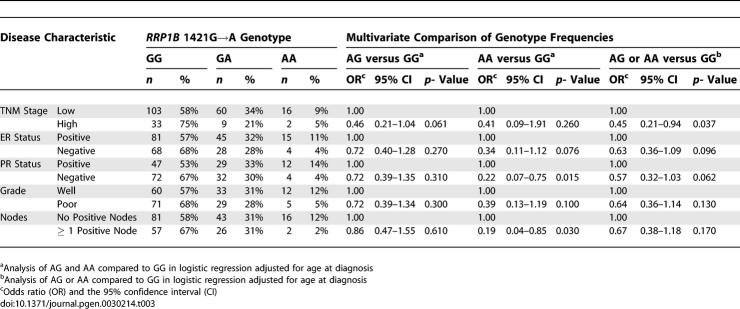
Association of Tumor Characteristics with the *RRP1B* 1421G→A SNP in the Baltimore Cohort

### Haplotype Analysis of the *RRP1B* Pro436Leu Variant

The Pro436Leu SNP was selected for analysis in the pilot epidemiology analysis due to its potential effect on *RRP1B* function as the result of the nonsynonymous amino acid substitution. The pilot epidemiology data, while consistent with the possibility of role of *RRP1B* in breast cancer progression, does not distinguish between causal polymorphisms and polymorphisms in high linkage disequilibrium (LD) with the causal variant. To gain a better understanding of whether the Pro436Leu SNP might be the causal variant effecting *RRP1B* function, haplotype analysis was performed using the publicly available HapMap data (http://www.hapmap.org/downloads/index.html.en). rs9306160 was genotyped on the 30 CEPH trios (90 samples) used in the HapMap project and the linkage disequilibrium (LD) structure determined. Analysis of the current version of the HapMap revealed that the Pro436Leu SNP was in a large haplotype block spanning 212,658 bp, with 40 SNPs in high LD with rs9306160 (r^2^ > 0.8). The haplotype block encompasses only two genes, RRP1B and HSF2BP. Among the 673 SNPs in this region rs9306160 is the only missense polymorphism within this haplotype block based on RefSeq annotation (http://www.ncbi.nlm.nih.gov/RefSeq/), and therefore remains an interesting candidate for the causative polymorphism. However, we cannot exclude at this time the possibility that the causative effect is due to a different linked polymorphism. Further analysis at the epidemiology and molecular level will be required to resolve this question.

## Discussion

The diverse techniques employed in this study, including genetical genomics [21], functional genomics, sequence analysis, and molecular epidemiology, have allowed us to identify *Rrp1b* as a candidate for both a tumor progression and metastasis modifier in mice, and a marker of inherited breast cancer metastasis susceptibility in humans. Furthermore, functional data provides evidence that *Rrp1b* regulates ECM gene expression, and that a nonsynonymous SNP in *RRP1B* is associated with tumor progression and disease-specific survival in pilot epidemiology experiments.

*Rrp1b* was identified through two distinct experimental approaches designed to address two independent questions: (a) what is the molecular mechanism(s) by which *Sipa1* modulates metastasis, and (b) what drives ECM dysregulation in metastasis-prone primary tumors? Yeast two-hybrid assays and functional genomic studies addressed the first question, which identified Rrp1b as binding to the polymorphic PDZ domain of Sipa1. The second question probed the origins of metastasis-predictive gene expression signatures. ECM genes are components of all metastasis-predictive gene expression signatures in both humans [9–11,22] and mice [12,13], a finding that may well be explained in part by constitutional variation [13]. To test this question, we examined whether ECM eQTLs and metastasis modifiers might be the same entities by analyzing ECM gene expression in a RI mouse panel. This led to the identification of several eQTLs, with a locus on proximal chromosome 17 displaying the strongest linkage. The peak linkage region of this locus encompasses both *Rrp1b* and a tumor growth and progression QTL [6], and rather remarkably, when we examined expression of transcripts within the peak eQTL linkage region, *Rrp1b* was highly correlated with metastasis-predictive ECM gene expression. Taken together, these data suggest that *Rrp1b* is a potential dual ECM and tumor progression candidate.

Further experimentation demonstrated that ectopic expression of *Rrp1b* in two highly metastatic mammary tumor cell lines and a mouse fibroblast cell line modulates ECM gene expression, a finding concurrent with our initial hypothesis that *Rrp1b* is indeed the chromosome 17 ECM eQTL. It should be noted however, that the directionality of the ECM expression changes observed in response to *Rrp1b* activation in these cell lines are not directly comparable to the changes in ECM expression observed in metastasis-predictive gene expression signatures [9–13]. Primary tumors are composed of a variety of cell and tissue types, and this cellular and microenvironmental heterogeneity is not accurately reflected by in vitro growth conditions of single cell types. Nevertheless, we do argue that our in vitro experimentation demonstrates that *Rrp1b* modulates the expression of metastasis-predictive ECM genes in a variety of individual cell lines. It is also evident that further work will be necessary to define the complex microenvironmental relationships that modulate ECM gene expression in bulk tumor tissue and their relationship to overall levels of *Rrp1b* activation.

In addition, it should be noted that eQTL analysis is most commonly associated with expression changes in normal tissues, and not the neoplastic tissues analyzed in this study. However, eQTLs owe their existence to germline polymorphism, and such variation will be present in tumor tissue in addition to normal tissues. With this in mind, it is therefore not unreasonable to assume that the phenotypic effects of eQTLs will be observed in tumors as well as normal tissue. Yet neoplastic tissues possess an inherent genomic instability, and it is therefore possible that variations in tumor gene expression patterns could also arise from somatic mutation. However, we have demonstrated that eQTLs can be genetically mapped in tumors, which suggests either that similar somatic mutations consistently occur in the majority of the tumor tissue in a subset of the RI strains, or that the observed eQTLs result from inherited polymorphisms. Our previous demonstration that the same ECM genes used to define tumor eQTLs are differentially expressed in normal mammary tissues derived from high- and low-metastatic mouse genotypes [13] suggests that such differential expression may be partially regulated by germline polymorphism. At this stage, however, we cannot formally dismiss a role for somatic mutation in ECM gene expression variation within RI mammary tumors.

We also described a number of important functional differences in *Rrp1b* between the progenitors of the AKXD RI panel, the high metastatic AKR/J, and low metastatic DBA/2J genotype mice. Specifically, the proximal promoter of *Rrp1b* in AKR/J mice contained polymorphisms that may reduce *Rrp1b* expression. This difference in promoter activity is one possible explanation for the observed differences in *Rrp1b* expression in the normal mammary tissue of these mice, with the high metastatic capacity AKR/J mouse having significantly lower levels of *Rrp1b* activity than the low metastatic DBA/2J mouse. Furthermore, ectopic expression led to reduced metastatic potential and primary tumor growth following tumor cell implantation into mice. In combination, these observations suggest, at least in this mouse model that increased *Rrp1b* expression correlate with a better outcome.

We have used a dual approach to try to address the importance of *RRP1B* in human breast cancer progression. The first of these approaches was to use data derived from microarray expression analysis of the Mvt-1/*Rrp1b* cells to address one of the central goals of our research: the translation of experimental data from mouse models of human breast cancer into potentially clinically relevant observations. Based on the work of Bild et al. [20] we identified an *RRP1B* gene expression signature and demonstrated it that predicts outcome in a publicly available and well-characterized breast cancer cohort [10]. This gene signature not only held strong prognostic value in the Dutch study cohort [10], but was also able to stratify those patients with ER positive tumors and LN negative disease at presentation into high and low risk categories. There has been significant interest in using gene expression profiles for improved patient stratification [23,24] in the clinic since it raises the possibility of improvements in breast cancer subtype classification, which in turn could enable clinicians to tailor treatment to individual patients. Whether the *RRP1B* signature proves of clinical value in this respect is at present unclear, and further testing of its prognostic value in different cohorts will be required to address this possibility. The significance of this study, however, is not the identification of yet another prognostic signature, but the fact that the underlying casual element is known. Identification of other genetic elements that drive the predictive gene expression patterns may provide a more robust means of complementing currently available tests used for the assessment of prognosis in breast cancer. Furthermore, this type of study also provides us with potentially novel and important insights into the mechanisms underlying the metastatic process.

Further supportive evidence for the role of *RRP1B* in human breast cancer progression was evident in the two pilot epidemiological studies, both of which found an inverse relationship between the variant A allele of the 1421G→A *RRP1B* SNP and poor outcome markers. These consistent findings indicate that 1421G→A is a marker for disease progression, and patients who carry the A allele are less likely to present with advanced disease than homozygous carriers of the more common G allele. These data are consistent with the results of functional analysis of *Rrp1b*, and associate *RRP1B* with disease outcome in human breast cancer. It should be noted that the variant 1421G→A allele was associated with improved outcome in those individuals with ER positive tumors, which may permit better stratification of patients who are currently thought to be in a low risk category. A particularly intriguing question is if and how a patient's 1421G→A genotype affects expression of the 33-gene *RRP1B* expression signature, and whether polymorphisms in the promoter of *RRP1B* in linkage disequilibrium with the 1421G→A SNP are more important in this respect. These studies are currently ongoing in this laboratory. Indeed, a link between constitutional polymorphism and bulk tumor gene expression would be particularly significant given the technical difficulties associated with tumor gene expression profiling and the relative ease of SNP genotyping. These results, while consistent between the studies and in support of our hypothesis, must be considered only as preliminary. Further investigations in larger epidemiology studies specifically designed to address tumor progression and outcome, rather than tumor incidence, will be necessary to gain further support for the role of germline polymorphism in *RRP1B* in breast cancer progression.

Several differences were evident between the epidemiology study cohorts, the most notable of which was that the 1421G→A SNP was associated with breast cancer-specific survival only in the Baltimore cohort, a discrepancy that is likely due to several factors. First, both cohorts are relatively small (*n* < 300), thus some differences might arise from statistical power issues. More importantly, the study population compositions differed: the Orange County cohort was derived from a population-based case-cohort study, including all cancer patients, regardless of stage, with more than 10 years of follow up, whereas the Baltimore cohort is a surgical breast cancer population, and therefore is biased against patients with metastatic disease at presentation. Furthermore, unlike the Orange County cohort, the Baltimore cohort contained African-American and Caucasian women. Differences in race/ethnicity influence allele frequency and disease outcome, and some variability in results is therefore expected. While the participation of patients from different race/ethnicities also strengthens study design, stratification is required. Stratification on race/ethnicity in the Baltimore cohort indicated that race/ethnicity was not a significant confounding factor. It is interesting to note, however, that the variant allele distribution is different in African-Americans and Caucasians in the Greater Baltimore cohort (0.099 in African-Americans versus 0.411 in Caucasians). It is known that African-American women have a poorer prognosis compared to other breast cancer patients [25], and given the protective effect exerted by the variant 1421G→A, it is interesting to speculate that polymorphisms in genes such as *RRP1B* may be driving these ethnicity-specific differences in outcome. Thus, further characterization of this SNP in larger and ethnically diverse cohorts is required to determine the influence of race/ethnicity upon the association between this SNP and breast cancer survival.

The function of *Rrp1b* and its exact role in metastasis remain unclear at this time. *Sipa1* was originally cloned as a mitogen-inducible protein [26] that was subsequently shown to be a negative regulator of Rap1 by serving as a GAP for Rap1 [27]. *Sipa1* has significant effects on cellular adhesion [28], primarily related to its effects on Rap1, which has been implicated in maintaining the integrity of polarized epithelia [29] and intercellular adherens junctions [30], and potentially integrating signaling between cadherins and integrins [31]. Rrp1b may therefore mediate tumor cell adhesion properties by altering intercellular and cell–ECM contacts in a Sipa1-dependent and Rap1-dependent manner. It should be noted, however, that the human polymorphism in *RRP1B* falls outside of the domain that directly interacts with the PDZ domain of Sipa1. Whether this polymorphism impacts the enzymatic function of Sipa1 or mediates metastatic potential through some other mechanism is unclear and currently under investigation. Similarly, it is unclear whether the amino acid substitution in human RRP1B directly affects function. Based on the mouse model, where increased expression confers protection against malignant progression, the variant leucine in the human ortholog may phenocopy the mouse situation by activating some function of *RRP1B.* Further in vitro analysis will be required to clarify the different situations in the two species and is currently under investigation in our laboratory.

In addition to the negative regulatory role on *Sipa1* function, there is some evidence to suggest that *Rrp1b* may be involved in RNA metabolism. Protein homology analysis has shown Rrp1b to contain a Nop52 domain, a motif found in proteins critical to 28S rRNA generation [32], and a previous yeast two-hybrid analysis has shown that Rrp1b may also interact with Lsm1, which is a protein involved in regulation of mRNA degradation [33]. Publicly available databases show that *RRP1B* is ubiquitously expressed at a somewhat low level, although it is expressed at a slightly higher level in lymph nodes in humans (http://smd.stanford.edu/cgi-bin/source/sourceSearch). Differential expression of *RRP1B* has been reported in fibroblasts from patients with systemic sclerosis, an autoimmune disorder characterized by dysregulation of a variety of ECM genes, including procollagens I, III, and VI [34], consistent with our results. Further research, however, is clearly needed to fully explore the role of *Rrp1b* and *Sipa1* in human breast cancer and other tumor types. Unraveling the mechanisms of action and the molecular pathways that they regulate are likely to provide novel and valuable insights into tumor dissemination and metastasis.

## Materials and Methods

### Yeast two-hybrid analysis.

Yeast two-hybrid screens using different regions of the human Sipa1 protein (Entrez Gene ID No: 6494) as bait were performed by ProNet technology (Myriad Genetics, Salt Lake City, UT). Methodology for these commercially performed experiments is provided by Myriad Genetics and is available in Text S2.

### Coimmunoprecipitation of Rrp1b with Sipa1.

The various genes were cloned into pcDNA3. HEK293 (293) cells were transiently transfected with lipofectamine (Invitrogen) according to the manufacturer's instructions. They were cotransfected with pcDNA3 vector or mouse Sipa1-V5 from DBA [7], and Rrp1b-V5, Rrp1b-HA, or AQP2 (from American Type Culture Collection). Two days after transfection, cells were lysed with Golden Lysis Buffer (GLB) containing 20 mM Tris (pH 7.9), 137 mM NaCl, 5 mM EDTA, 1 mM EGTA, 10 mM NaF, 10% Glycerol, 1% Triton X-100, 1 mM sodium pyrophosphate, 1 mM leupeptin, 1 mM PMSF, and aprotinin (10 μg/ml). Cell extracts were immunoprecipitated with anti-Spa-1 (Sipa1) mAb (BD BioSciences), and protein A/G Sepharose (Pierce) were added and rotated overnight at 4 °C. The immune complexes were washed once with GLB, once with high salt HNTG (20 mM Hepes, 500 mM NaCl, 0.1% Triton X-100, 10% Glycerol), and twice with low salt of HNTG (20 mM Hepes, 150 mM NaCl, 0.1% Triton X-100, 10% Glycerol). The immune complexes were then analyzed by immunoblotting with antibodies against AQP2 (Santa Cruz), V5 (Invitrogen), or HA (Convance). For each immunoblot, horseradish peroxidase-conjugated anti-rabbit, anti-mouse, or anti-goat immunoglobulin G was used for the second reaction at a 1:10,000 dilution. Immunoblots were visualized by enhanced chemiluminescence with an ECL Kit (Amersham).

### RALGDS pull-down assay.

293 cells were transiently cotransfected with pcDNA3 vector or mouse Sipa1 and Rrp1b-V5 or AQP2. Epac-HA was also cotransfected, to elevate the level of Rap1-GTP, and pcDNA3 vector was added as necessary to ensure that equal amounts of DNA were transfected. Transfected cells were processed two days later, using a Rap1 activation Kit (Upstate Biotech) according to the manufacturer's instructions. Equal amounts of the total protein from cell extracts were estimated based on BCA protein assay kit (Pierce). Rap1-GTP protein was pulled down by RalGDS-RBD beads and washed three times, then subjected to gel analysis and immunoblotting using anti-Rap1 antibody (Santa Cruz). Cell extracts from transfectants were analyzed for protein expression by immunoblotting, using anti-AQP2 antibody, anti-Spa-1 mAb, or anti-V5 antibody.

### Cell culture.

The Mvt-1 and 4T1 cell lines were obtained as a gift from Lalage Wakefield (National Cancer Institute, Bethesda). The cells were cultured in Dulbecco's Modification of Eagle's Medium (DMEM; Cellgro, VA) containing 10% fetal bovine serum (FBS; Cellgro, VA), with culture medium being replaced at three day intervals. When the cells achieved confluency, they were washed once with 5 ml phosphate-buffered saline (PBS), incubated with 2 ml trypsin-EDTA for 5 min, and passaged at a 1:30 dilution into a fresh culture flask. NIH-3T3 cells were maintained in the same manner as Mvt-1, except cells were passaged at a 1:15 ratio when they achieved confluency.

### Expression QTL mapping.

Microarray hybridization methodology and generation of the microarray expression data from AKXD × PyMT primary tumors has been described previously [13]. Affymetrix .CEL files were normalized using the RMA method, averaged for each AKXD RI strain, and loaded into the GeneNetwork web service (http://www.genenetwork.org) [15]. The database was then searched for the 11 probe sets from our previously described metastasis signature profile [13] classification as within an “ECM component” (1437568_at, *Mmp16*; 1418454_at, *Mfap5*-pending; 1416168_at, *Serpinf1*; 1425896_a_at, *Fbn1*; 1416625_at, *Serping1*; 1450798_at, *Tnxb*; 1439364_a_at, *Mmp2*; 1427884_at, *Col3a1*; 1416808_at, *Nid1*; 1423407_a_at, *Fbln2*; and 1420924_at, *Timp2*). Additionally, a further five probe sets for the ECM genes represented the human breast carcinoma metastasis gene signature profile described by Ramaswamy et al. [9] were also included (*Col1a1*: 1423669_at_A, 1455494_at_A and *Col1a2*: 1423110_at_A, 1446326_at_B, 1450857_a_at_A). eQTLs were defined as described above.

### Development of Mvt-1 and 4T1 clonal isolates ectopically expressing *Rrp1b.*

An expression vector encoding the full length *Rrp1b* cDNA BC016569 in pCMV-SPORT6 was obtained from the Mammalian Gene Collection (MGC:27793, IMAGE ID: 3157173). The control cell line was generated using the vector pCMV-SPORT-β-Galactosidase (Invitrogen). The identity of the vector was sequence verified before transfection. Supercoiled plasmids were transfected into Mvt-1 and 4T1 cells using Superfect Transfection Reagent (Qiagen, Valencia, CA) as per the manufacturer's instructions. Briefly, transfections were performed in 100 mm diameter culture dishes, with 2 ×10^6^ Mvt-1 or 4T1 cells being seeded 24 h prior to transfection. The *Rrp1b*-pCMV-Sport6 and pCMV-SPORT-β-Galactosidase vectors were cotransfected with the vector pSuper.Retro.Puro (Oligoengine) containing no insert as a selectable marker for transfectants. Cells in each culture vessel were transfected with a total of 20 μg vector DNA using Superfect at a 6:1 lipid to DNA ratio. Twenty-four hours after transfection, the cells were selected in normal growth medium containing 10 μg/ml puromycin (Sigma Aldrich), transferred to 96 well plates, and individual clones were selected by limiting dilution. Colonies were screened by qPCR as described below to identify clones ectopically expressing *Rrp1b*.

With regards to the NIH-3T3 cell lines, PCR primers were designed to encompass the entire length of the *Rrp1b* cDNA BC016569. The following primer sequences generated a 2,248 bp product from normal mammary tissue cDNA, with the downstream primer being designed to omit the transcription termination codon and to remain in coding frame: 5′-CCCATACGCAGACGCAGT-3′ and 5′-GAAGAAGTCCGCAGCCCT-3′. Full length *Rrp1b* cDNA was then amplified using rTth DNA Polymerase, XL (Applied Biosystems) as per the manufacturer's protocol. Following PCR amplification, *Rrp1b* cDNA was inserted into the reporter vector pcDNA3.1 V5-His using a pcDNA3.1 V5-His TOPO® Cloning Kit (Invitrogen) and transformed into TOP10 competent cells (Invitrogen) as per the manufacturer's protocol. Plasmids were propagated in 100 ml LB Medium containing 100μg/ml ampicillin. Plasmid DNA was extracted using a Qiagen EndoFree Maxi Kit, and insert identity and integrity of the full-length sequence was verified prior to further experimentation. Transfection of this vector into NIH-3T3 cells was performed in the same manner as for Mvt-1 cells except that the *Rrp1b* construct was cotransfected with the puromycin selection marker pPur (Clontech), and selection of transformants performed using 5 μg/ml puromycin and 700 μg/ml G418 (Sigma Aldrich).

### Total RNA isolation for qPCR.

Total RNA samples were isolated from cell culture samples using an RNeasy Mini Kit (Qiagen) with sample homogenization being performed using a 21 gauge needle and syringe as per the manufacturer's protocol. All samples were subjected to on-column DNase digestion, and RNA quality and quantity determined by an Agilent Technologies 2100 Bioanalyzer (Bio Sizing Software version A.02.01, Agilent Technologies). Only those samples containing high-quality total RNA with A_260_/A_280_ ratios between 1.8 and 2.1 were used for further analysis.

### Quantitative Real-Time PCR gene expression analysis.

cDNA was synthesized from RNA isolated from either primary tumor tissues or transfected cell lines using the ThermoScript RT-PCR System (Invitrogen, Carlsbad, CA) by following the manufacturer's protocol. Single RT-PCRs were performed for each Mvt-1 and 4T1 clonal isolates, and in triplicate for the untransfected or *Rrp1b*-expressing NIH-3T3 total RNAs. SYBR Green qPCR was performed to detect the cDNA levels of *Rrp1b* and a variety of metastasis predictive ECM genes (see above) using an ABI PRISM 7500 and/or 7900HT Sequence Detection Systems and custom designed primers (Table S12). Reactions were performed using QuantiTect SYBR Green Master Mix (Qiagen, Valencia, CA) as per the manufacturer's protocol. The cDNA level of each gene was normalized to *peptidylprolyl isomerase B* (*Ppib*) cDNA levels using custom-designed primers for SYBR green-amplified target genes (Table S12).

### Sequencing of the *Rrp1b* gene.

Complete sequencing of the exons, intron-exon boundaries, promoters, and the regions immediately upstream of the promoters was performed in two highly metastatic (AKR/J, FVB/NJ) and three low metastatic (DBA/2J, I/LnJ, NZB/B1NJ) strains of mice [35]. The sequences of the primers for *Rrp1b* are shown in Table S4. PCR products were generated under standard amplification conditions (5 min at 94 °C, 30 s at 57 °C, 30 s at 72 °C, and 5 min at 72 °C), purified with a Qiagen PCR purification kit, and double strand sequencing performed with a Perkin Elmer BigDye Terminator sequence kit. Analysis was performed on a Perkin Elmer 3100 Automated Fluorescent Sequencer. Sequences were compiled and analyzed with the computer software package VectorNTI [36].

### Generation of *Rrp1b* proximal promoter β-galactosidase reporter constructs.

PCR primers were designed to encompass the two proximal promoter polymorphisms identified by sequencing *Rrp1b*. The following primer sequences generated a 1,672 bp product with AKR/J genomic DNA and a 1,670 bp product with DBA/2J: 5′-AACCTCATCGTCCCTTGG-3′ and 5′-GCACTCGCTTCAGCATCC-3′. Proximal promoter sequences were amplified using rTth DNA Polymerase, XL (Applied Biosystems) as per the manufacturer's protocol. Following PCR amplification, proximal promoter sequences were inserted into the reporter vector pBlue TOPO using a pBlue TOPO® TA Expression Kit (Invitrogen) and transformed into TOP10 competent cells (Invitrogen) as per the manufacturer's protocol. Plasmids were propagated in 100 ml of Luria-Bertani Medium containing 100 μg/ml ampicillin. Plasmid DNA was extracted using a Qiagen EndoFree Maxi Kit. AKR/J and DBA/2J promoter constructs were sequence verified prior to further experimentation as described above.

### Quantification of activity of *Rrp1b* proximal promoter β-galactosidase reporter constructs.

Supercoiled plasmids were transfected into NIH-3T3 cells using Superfect Transfection Reagent (Qiagen, Valencia, CA) as per the manufacturer's instructions. Briefly, transfections were performed in 100 mm diameter culture dishes, with 2 × 10^6^ NIH-3T3 cells seeded 24 h prior to transfection with 10μg vector DNA (8μg *Rrp1b* promoter construct and 2μg of pGL3-Control (Promega)) using Superfect at a 5:1 lipid to DNA ratio. Transfections were performed in triplicate for each promoter construct. Twenty-four hours after transfection, the cells were washed with PBS, trypsinized, collected, and washed twice with cold PBS. Cell lysis was achieved using 100 μl of lysis buffer per 100 ml plate from a β-Galactosidase Assay Kit (Invitrogen). β-galactosidase activity in each sample was assayed as per the manufacturer's protocol using either 20 μl or 30 μl of the lysate. The remaining lysate was used to determine lysate protein concentration, which was assayed using a BCA Assay Kit (Pierce). β-galactosidase activity was calculated for each sample as per the β-Galactosidase Assay Kit (Invitrogen), but is essentially based upon the concentration and A_562_ of each lysate. These specific activities were normalized against firefly luciferase activity as driven by the pGL3 control vector to account for differing transfection efficiencies. The activity of firefly luciferase for each sample was assayed using a Dual-Luciferase Reporter Assay Kit (Promega) and quantification performed by VICTOR2 (Perkin-Elmer Life Sciences).

### Quantification of *Rrp1b* expression in normal mammary tissue.

Total RNA extractions from tissue samples were carried out using TRIzol^®^ Reagent (Life Technologies, Inc.) according to the standard protocol. RNA quantity and quality were determined by the Agilent Technologies 2100 Bioanalyzer (Bio Sizing Software version A.02.01, Agilent Technologies) and/or the GeneQuant Pro (Amersham Biosciences). Samples containing high-quality total RNA with A_260_/A_280_ ratios between 1.8 and 2.1 were purified with the RNeasy Mini Kit (Qiagen). An on-column genomic DNA digestion was performed as part of this purification step using the RNase-Free DNase Kit (Qiagen). TaqMan qPCR was performed to detect the cDNA levels of *Rrp1b* using an ABI PRISM 7500 Sequence Detection System (Applied Biosystems, Foster City, CA). *Rrp1b* expression was quantified using the Applied Biosystems Assay-On-Demand Mm00551206_m1. The housekeeping gene *Peptidylprolyl Isomerase B* (*Ppib*), used for normalization of *Rrp1b* expression between samples, was quantified using the primers described in Table S12 and the following fluorogenic probe: 6FAM-TCTATGGTGAGCGCTTC-MGB. Reactions were performed using TaqMan Universal PCR Mastermix (Applied Biosystems) per the manufacturer's protocol.

### Spontaneous metastasis assays.

Transfected cells proven to be stably expressing *Rrp1b* were subcutaneously implanted into virgin FVB/NJ mice. Two days before injection, cells were passaged and permitted to grow to 80%–90% confluence. The cells were then washed with PBS and trypsinized, collected, washed twice with cold PBS, counted in hemocytometer, and resuspended at 10^6^ cells/ml. One hundred thousand cells (100 μl) were injected subcutaneously in the vicinity of the fourth mammary gland of 6-wk-old virgin FVB/NJ female mice. The mice were then aged for 4 wk before they were killed by anesthetic overdose. Tumors were dissected and weighed. Lungs were isolated and surface metastases enumerated using a dissecting microscope. Tumor growth and metastasis was compared to mice injected with 10^5^ Mvt-1 cells stably cotransfected with pCMV-Sport-β-Gal and pSuper.Retro.Puro. These experiments were performed in compliance with the National Cancer Institute's Animal Care and Use Committee guidelines.

### SNP genotyping.

The *Rrp1b* 1421G→A polymorphism was characterized using SNP-specific PCR. PCR primers were designed using Primer Express software (Applied Biosystems) according to parameters described elsewhere [37]. Each probe was labeled with a reporter dye (either VIC^®^, a proprietary fluorescent dye produced by Applied Biosystems, or FAM, 5-(&6)-carboxyfluorescein) specific for the wild-type and variant alleles of the *Rrp1b* SNP. Sequences of PCR primers are as follows: 5′-TGGACGTGGCCTCTGCAC-3′ and 5′-CACCACCTGCAGCCTGAAA-3′; and the sequences of fluorogenic probes are as follows: 6FAM-AGGGCTTTCAGCCCAGAG and VIC-AGGGCTTTCGGCCCAG. Reaction mixtures consisted of 300 nM of each oligonucleotide primer, 100 nM fluorogenic probes, 8 ng template DNA, and 2× TaqMan Universal PCR Master Mix (Applied Biosystems, Foster City, CA) in a total volume of 10 μl. The amplification reactions were performed in a MJ Research DNA Engine thermocycler (Bio-Rad, Hercules, CA) with two initial hold steps (50 °C for 2 min, followed by 95 °C for 10 min) and 50 cycles of a two-step PCR (92 °C for 15 s, 60 °C for 1 min). The fluorescence intensity of each sample was measured post-PCR in an ABI Prism 7500 sequence detection system (Applied Biosystems, Foster City, CA), and *Rrp1b* SNP genotypes were determined by the fluorescence ratio of the nucleotide-specific fluorogenic probes.

### RNA extraction and processing for Affymetrix GeneChip analysis

Total RNA was extracted using TRIzol Reagent (Life Technologies) according to the standard protocol. Total RNA samples were subjected to DNase I treatment, and sample quantity and quality determined as described above. Purified total RNA for each clonal isolate were then pooled to produce a uniform sample containing 8 μg of RNA.

Double stranded cDNA was synthesized from this preparation using the SuperScript Choice System for cDNA Synthesis (Invitrogen) according to the protocol for Affymetrix GeneChip Eukaryotic Target Preparation. The double stranded cDNA was purified using the GeneChip Sample Cleanup Module (Qiagen). Synthesis of biotin-labeled cRNA was obtained by in vitro transcription of the purified template cDNA using the Enzo BioArray High Yield RNA Transcript Labeling Kit (T7) (Enzo Life Sciences). cRNAs were purified using the GeneChip Sample Cleanup Module (Qiagen). Hybridization cocktails from each fragmentation reaction were prepared according to the Affymetrix GeneChip protocol. The hybridization cocktail was applied to the Affymetrix GeneChip Mouse Genome 430 2.0 arrays, processed on the Affymetrix Fluidics Station 400, and analyzed on an Agilent GeneArray Scanner with Affymetrix Microarray Suite version 5.0.0.032 software. Normalization was performed using the BRB-Array Tools software [38,39].

### Microarray and survival analysis.

To generate a high confidence human transcriptional signature of *Rrp1b* expression, 98 probe sets whose differential expression demonstrated *p* < 10^−7^ were selected by matching the gene symbols from the mouse dataset to the published Hu25K chip annotation files. Analysis of tumor gene expression from breast cancer datasets was performed using BRB ArrayTools. Expression data were downloaded from the Rosetta Company website (http://www.rii.com/publications/2002/vantveer.html). Expression data were loaded into BRB ArrayTools using the Affymetrix GeneChip Probe Level Data option or the Data Import Wizard. Data were filtered to exclude any probe set that was not a component of the *Rrp1b* signature, and to eliminate any probe set whose expression variation across the data set was *p* ≥ 0.01.

Unsupervised clustering of each dataset was performed using the Samples Only clustering option of BRB ArrayTools. Clustering was performed using average linkage, the centered correlation metric and center the genes analytical option. Samples were assigned into two groups based on the first bifurcation of the cluster dendogram, and Kaplan-Meier survival analysis performed using the Survival module of the software package Statistica. Significance of survival analyses was performed using the Cox F-test.

### Breast cancer cohorts.

*Orange County, California.* This patient population is a random sample of 269 probands successfully genotyped for the Rrp1b SNP that were diagnosed between March 1, 1994 and February 28, 1995 with invasive breast cancer. Probands were ascertained through the population-based Hereditary Breast Cancer Study funded by the National Cancer Institute. A description of the study and details of data collection methods have been reported previously [8,40]. Briefly, there were two case groups: cases with localized disease (*n* = 130), and cases with regional/metastatic disease (*n* = 139). The average age at diagnosis of this cohort was 55.9 ± 13.4 years, and the average body mass index was 25.7 ± 4.9.

*Greater Baltimore, Maryland.* Surgical breast cancer cases were recruited at the University of Maryland Medical Center, the Baltimore Veterans Affairs Medical Center, Union Memorial Hospital, Mercy Medical Center, and the Sinai Hospital in Baltimore, Maryland between February 15, 1993 and August 27, 2003. We collected blood, tissue specimens, and survival information from 248 patients. These patients had pathologically confirmed breast cancer, were of African-American or Caucasian descent by self-report, were diagnosed with breast cancer within the last six months prior to recruitment, and had, by self-report, no previous history of the disease. Patients were excluded if they were HIV, hepatitis B virus, or hepatitis C virus carriers, were intravenous drug users, were institutionalized, or were physically or mentally unable to sign consent and complete the questionnaire. Of the eligible patients that were identified through surgery lists, 83% participated in the study. The subjects signed a consent form and completed an interviewer-administered questionnaire. Additional information to determine the ER-α status, disease stage, treatment, and survival was obtained from medical records and pathology reports, the Social Security Death Index, and the National Death Index. Disease staging was performed according to the TNM system of the American Joint Committee on Cancer/ the Union Internationale Contre le Cancer (AJCC/UICC). The Institutional Review Boards at the participating institutions approved the study.

### Statistical analysis.

Student's t-tests were used to compare means for continuous variables and Wilcoxon's sum rank test to compare medians. Variables that were not normally distributed, such as tumor size, were log transformed. Chi-square test or Fisher's exact tests were used to test for differences between categorical variables and to test for Hardy-Weinberg equilibrium. Unconditional logistic regression adjusting for multivariate covariates, such as age at diagnosis, was used to estimate the adjusted odds ratios. We used likelihood ratio tests to calculate *p-*values comparing a model with covariates to a model without them. We used Cox regression models to perform survival analysis. All *p-*values presented are two-tailed and were considered to be statistically significant if they were below 0.05. The Kaplan-Meier method and the log-rank test were used for univariate survival analysis and Cox regression models were used to perform multivariate survival analysis. For the Baltimore cohort, survival was determined for the period from the date of hospital admission to the date of the last completed search for death entries in the Social Security Index (August 18, 2004) for the 248 case patients. The mean follow-up time for breast cancer survival was 55 months (range: 12 to 140 months). A total of 59 (24%) of these 248 patients died during this period. We obtained death certificates of the deceased case patients and censored all causes of death that were not related to breast cancer, such as accidents, violent crimes, stroke, heart attack, and liver cirrhosis, in our analysis.

### Haplotype analysis.

**Figure 6:**
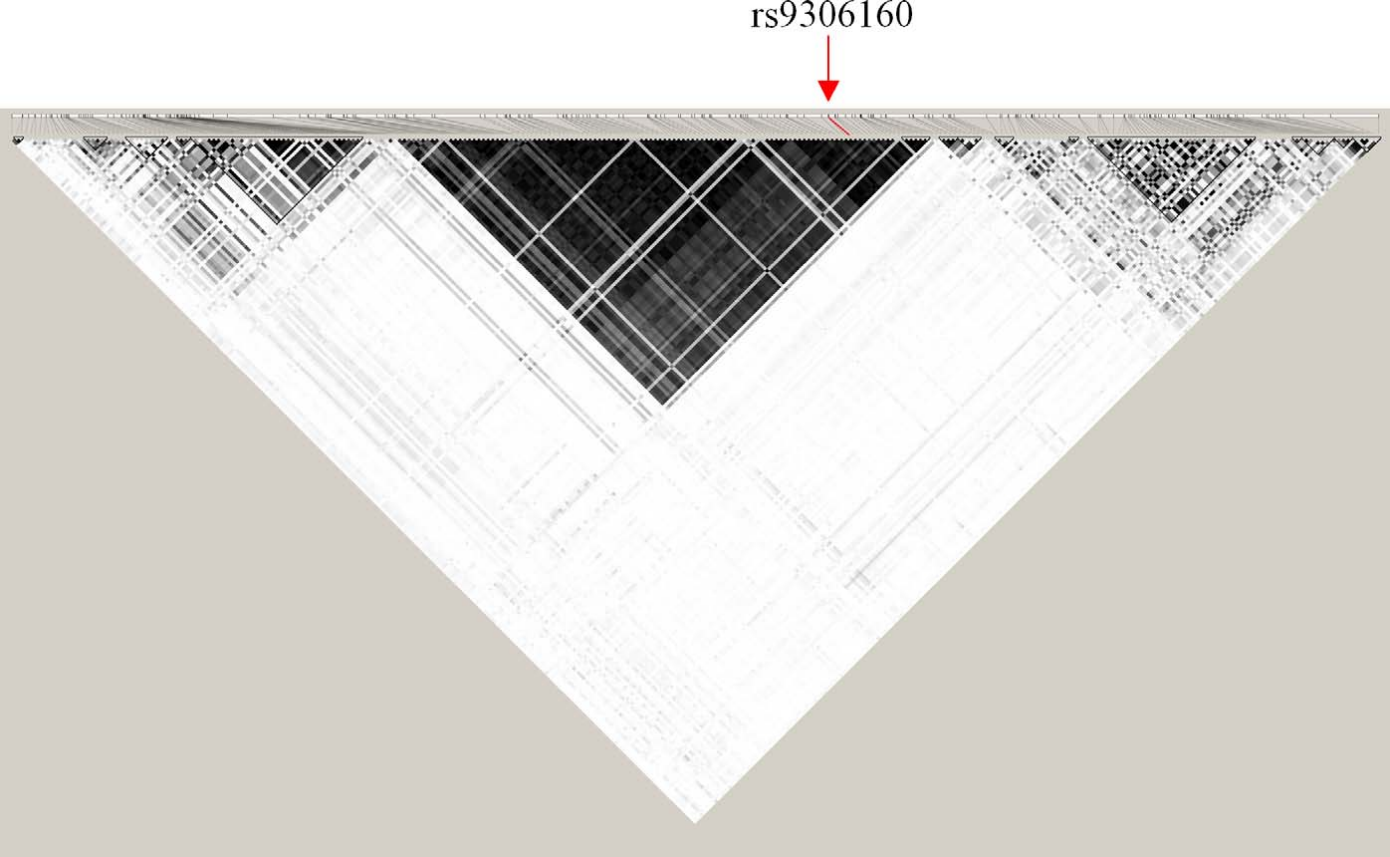
Haplotype Structure Surrounding rs9306160 in the CEPH Families The LD structure of rs9306160. Pairwise r^2^ values are shown with gray scale; the higher the r^2^ value the darker the color. The red line and arrow mark the location of rs9306160. The large block in the middle represents the 210 kb LD block where 104 SNPs are in high LD with rs9306160. The figure was generated by the program HaploView [42].

The genotype data of the 90 HapMap CEPH trio in the 250 kb flanking region of rs9306160 was downloaded from the HapMap database (http://www.hapmap.org). A total of 304 SNPs with minor allele frequency ≥10% were selected to evaluate the LD structure. The CEPH genotype data we generated for rs9306160 were integrated with the HapMap data in this analysis. Using LDSelect [41] with a cut of r^2^ ≥ 0.64 we found a large LD block spanning a 210 kb genomic region (chr21:43739961–43952618) that has 104 SNPs in high LD with rs9306160 (Figure 6). In this 210 kb region, there are a total of 673 dbSNP markers and rs9306160 is the only nonsysnonymous SNP based on RefSeq annotation.

## Supporting Information

Figure S1 Chromosome 17 ECM eQTL Locus in AKXD RI Mice ECM eQTL analysis in AKXD mice revealed that a locus on proximal chromosome 17 influenced the expression of metastasis-predictive ECM genes. The chromosome 17 locus peak linkage region (∼29.5 Mb) colocalizes with a previously described metastasis efficiency and tumor growth kinetics QTL [6], and encompasses the physical location of *Rrp1b* (∼29.9 Mb).
(255 KB PDF)

Figure S2 Mvt-1/*Rrp1b* Growth Curve Ectopic expression of *Rrp1b* does not alter the growth kinetics of the Mvt-1 cell line.
(265 KB PDF)

Figure S3 Strain-Specific Variation in *Rrp1b* Promoter Activity Promoter activity assays reveal that *Rrp1b* proximal promoter activity was reduced 30% in AKR/J genotype relative to its DBA/2J counterpart (*p* < 0.001).
(276 KB PDF)

Table S1 Baits Represented in Yeast-Two Hybrid SIPA1 Interaction Analysis (16 KB XLS)

Table S2 Proteins Interacting With SIPA1 on Yeast-Two Hybrid Interaction Analysis (19 KB XLS)

Table S3 Genes with Expression Associated with Metastasis-Predictive ECM Genes on eQTL Analysis: Chromosome 17 Locus (47 KB XLS)

Table S4 Rrp1b Sequencing Amplicons: Primers and Polymorphisms (25 KB XLS)

Table S5 Promoter Activity Assays for AKR/J and DBA/2J Proximal Rrp1b Promoters (30 KB XLS)

Table S6 qPCR Analysis of Rrp1b Expression in the Normal Mammary Tissue of AKR/J and DBA/2J Mice (21 KB XLS)

Table S7 Genes Upregulated by Ectopic Expression of Rrp1b in Mvt-1 Cells (142 KB XLS)

Table S8 Genes Downregulated by Ectopic Expression of Rrp1b in Mvt-1 Cells (181 KB XLS)

Table S9 Hazard Ratio for Genes Associated With Survival (17 KB XLS)

Table S10 Characteristics of the Orange County Breast Cancer Cohort (18 KB XLS)

Table S11 Characteristics of the Greater Baltimore Breast Cancer Cohort (18 KB XLS)

Table S12 Quantitative RT-PCR Primer Sequences (19 KB XLS)

Text S1 Supplementary Data A detailed explanation and discussion of the ECM eQTL data.
(76 KB DOC)

Text S2 Supplementary Methods (62 KB PDF)

### Accession Numbers

 Accession numbers for genes mentioned in this paper from the Mammalian Gene Collection (http://mgc.nci.nih.gov/) are *Rrp1b* cDNA BC016569 (MGC:27793, IMAGE ID: 3157173).

Accession numbers for genes mentioned in this paper from the Entrez Gene database (http://www.ncbi.nlm.nih.gov/sites/entrez?db=gene) are *β-galactosidase* (957271), *Col1a1* (12842), *Col3a1* (1281), *Col6a2* (12834), *Fbln2* (14115), *Fbn1* (14118), *Mfap5* (50530), *Ppib* (19035), *Rrp1b* (72462), *RRP1B* (23076), *Sipa1*(20469), *Serpinf1* (20317), and *Serping1*(12258).

Accession numbers for probe sets mentioned in this paper from the Affymetrix NetAffx Analysis Center (http://www.affymetrix.com/analysis/index.affx) are *Col3a1* (1427884_at), *Col1a1* (1423669_at_A, 1455494_at_A), *Col1a2* (1423110_at_A, 1446326_at_B, 1450857_a_at_A), *Fbn1*(1425896_a_at), *Fbln2* (1423407_a_at), *Mfap5*-pending (1418454_at), *Mmp2* (1439364_a_at), *Mmp16* (1437568_at), *Nid1* (1416808_at), *Serpinf1*(1416168_at), *Serping1* (1416625_at), *Timp2*(1420924_at), and *Tnxb* (1450798_at).

Accession number for the polymorphism mentioned in this paper from the Entrez SNP database (http://www.ncbi.nlm.nih.gov/sites/entrez?db=Snp) is *Rrp1b* (dbSNP ID: rs9306160; 1421G→A, Pro436Leu)

## Abbreviations

CI: confidence interval
ECM: extracellular matrix
Epac: Rap exchange factor
eQTL: expression quantitative trait locus
ER: estrogen receptor
GAP: GTPase activating protein
HR: hazard ratio of death
LD: linkage disequilibrium
LN: lymph node
LRS: likelihood ratio statistic
PR: progesterone receptor
PyMT: polyoma middle-T
qPCR: quantitative real-time PCR
RI: recombinant inbred
RT-PCR: reverse-transcription PCR
SNP: single nucleotide polymorphism
TNM: tumor–node–metastasis
