# Supplementary figures and images for: Rrp1b, a New Candidate Susceptibility Gene for Breast Cancer Progression and Metastasis
Source: PLoS Genet. 2007 Nov 30;3(11):e214. doi: 10.1371/journal.pgen.0030214 (PMC2098807; doi:10.1371/journal.pgen.0030214)

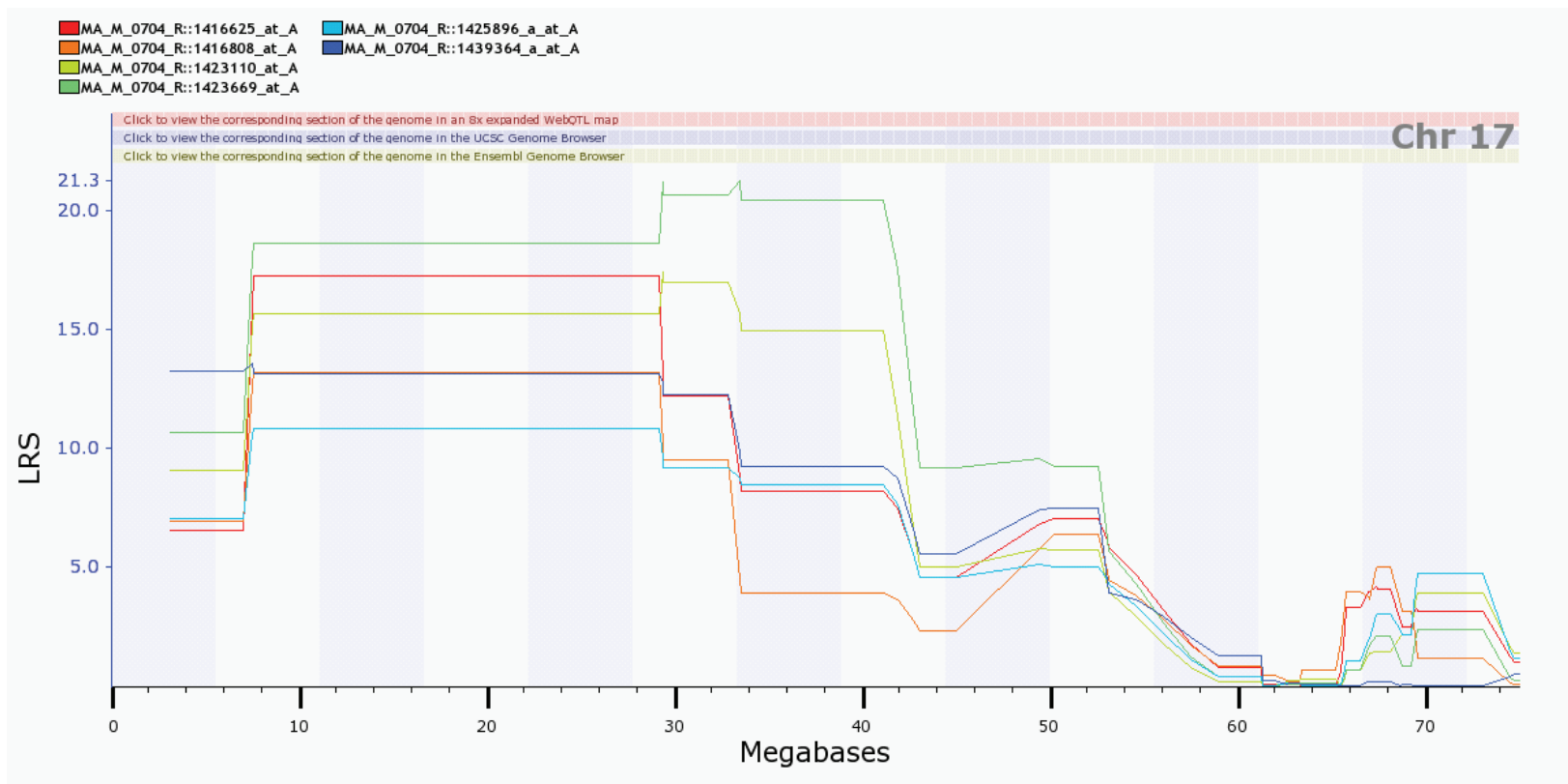

Supplement: Figure S1 — ECM eQTL analysis in AKXD mice revealed that a locus on proximal chromosome 17 influenced the expression of metastasis-predictive ECM genes. The chromosome 17 locus peak linkage region (∼29.5 Mb) colocalizes with a previously described metastasis efficiency and tumor growth kinetics QTL [6], and encompasses the physical location of Rrp1b (∼29.9 Mb). (255 KB PDF) [file pgen.0030214.sg001.pdf]

**Mvt-1/*Rrp1b* Growth Curve**

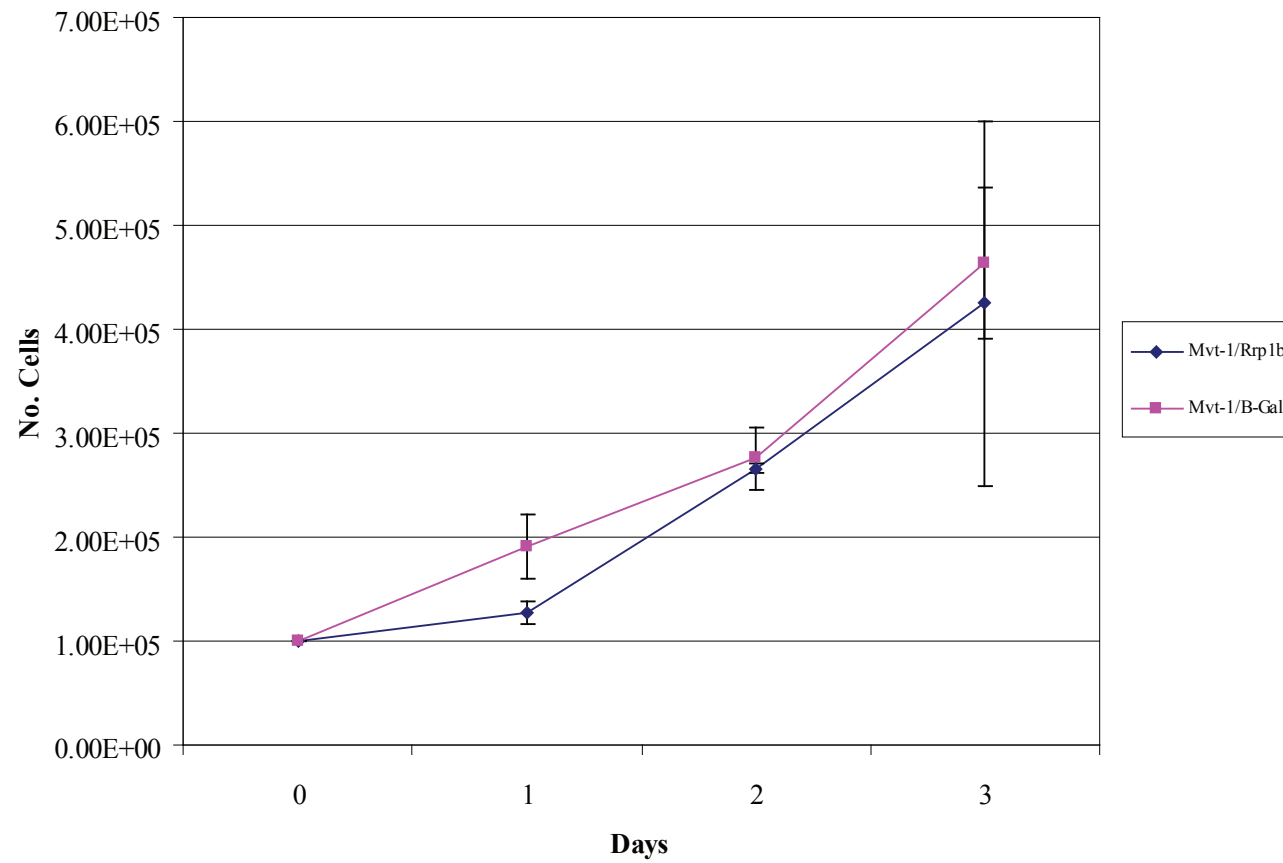

Supplement: Figure S2 — Ectopic expression of Rrp1b does not alter the growth kinetics of the Mvt-1 cell line. (265 KB PDF) [file pgen.0030214.sg002.pdf]

**Strain-Specific Variation in *Rrp1b* Promoter Activity**

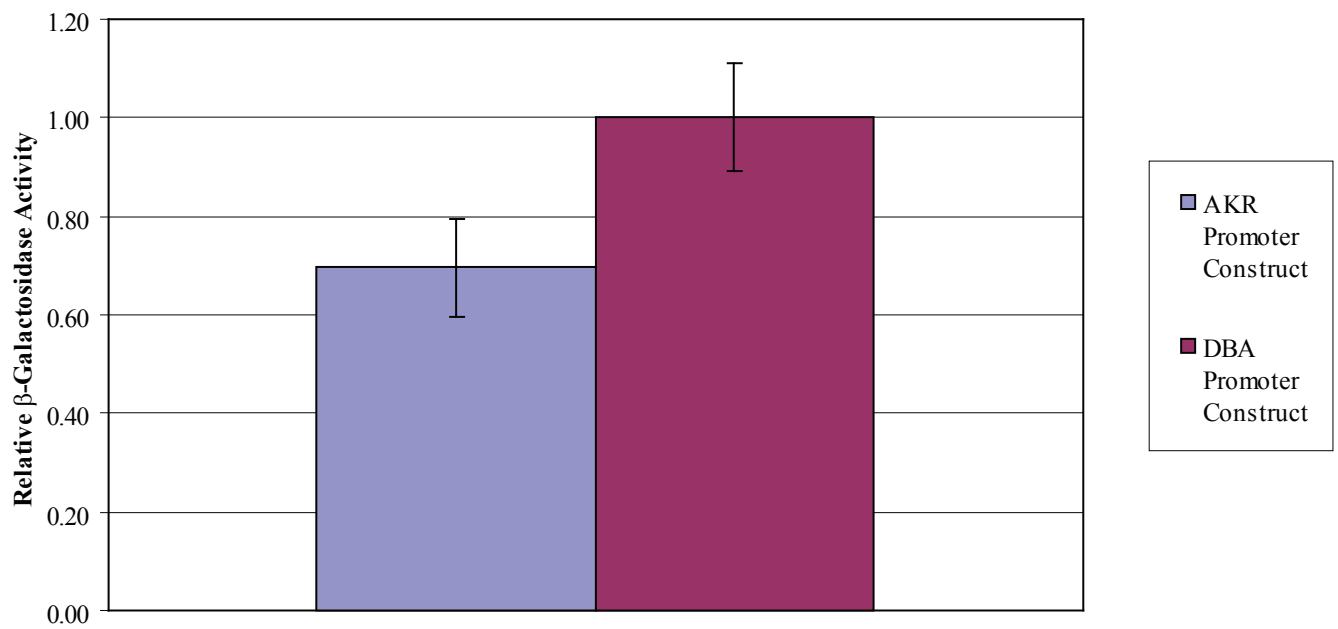

Supplement: Figure S3 — Promoter activity assays reveal that Rrp1b proximal promoter activity was reduced 30% in AKR/J genotype relative to its DBA/2J counterpart (p < 0.001). (276 KB PDF) [file pgen.0030214.sg003.pdf]
