# Supplementary material for: Rrp1b, a New Candidate Susceptibility Gene for Breast Cancer Progression and Metastasis
Source: PLoS Genet. 2007 Nov 30;3(11):e214. doi: 10.1371/journal.pgen.0030214 (PMC2098807; doi:10.1371/journal.pgen.0030214)
Supplement: Text S1 — A detailed explanation and discussion of the ECM eQTL data. (76 KB DOC) [file pgen.0030214.sd001.doc]

**Expression QTL mapping in AKXD recombinant inbred mice**

Expression profiling of AKXD RI mammary tumors was described previously[1]. Expression data was deposited on WebQTL[2], a publicly available internet-based analytical package/repository that allows for analysis of RI microarray expression data[3]. To map eQTLs correlating with ECM gene expression, probe sets were identified from 11 previously identified metastasis predictive genes ECM genes[1] (*Col3a1*, *Fbn1*, *Fbln2*, *Mfap5*, *Mmp2*, *Mmp16*, *Nid1*, *Serpinf1*, *Serping1*, *Timp2* and *Tnxb*). Additionally, five probe sets representing the two ECM genes present in the 17 gene human breast carcinoma metastasis gene signature profile described by Ramaswamy et al[4] were also included in the analysis (*Col1a1*, *Col1a2*). eQTLs for ECM probe sets were generated by identifying associations between genetic markers and probe intensities across the AKXD RI genetic mapping panel using the Interval Mapping function of WebQTL.

Extracellular matrix eQTL analysis was performed using the marker regression and interval mapping functions of WebQTL. Reproducible ECM eQTLs were discovered on chromosomes 7, 17 and 18 (Figure 2). The chromosome 17 locus was of particular interest as it co-localizes with a previously described metastasis efficiency and tumor growth kinetics QTL, with an observed approximate peak linkage at D17Mit81 (physical location ~ 29.1Mb)[5]. Significant eQTLs (genome-wide *P*<0.05) identified for two of the extracellular matrix genes used in the screen (*Col1a1*, *Fbn1*), located on proximal chromosome 17 (see Table 1 below and Supplementary Figure 1). Significance was determined by permutation. The 95% confidence interval (CI) for these genes overlapped and spanned roughly the first 40Mb of the chromosome (*Col1a1* 95% CI 8-42Mb; *Fbn1* 0-33Mb). 95% CIs were defined as a 2-LOD drop from the peak of the eQTL linkage.

**Table 1: Results of ECM eQTL screen**

| **Statistically significant eQTLs** | | |
| --- | --- | --- |
| Gene Symbol | Peak linkage (Mb*) | 95% CI (Mb*) |
|  |  |  |
| *Col1a1* | 30-41 | 8-42 |
| *Fbn1* | 0-7 | 0-33 |
|  |  |  |
| **Suggestive eQTLs** | | |
| Gene Symbol | Peak linkage (Mb*) | 95% CI (Mb*) |
|  |  |  |
| *Serping1* | 8-30 | 7-40 |
| *Col1a2* | 29-33 | 7-42 |
| *Nid1* | 7-29 | 0-31 |
| *Mmp2* | 0-32 | 0-54 |
| *Mfap5* | 7-30 | 0-58 |

*= megabase position from centromere

In addition to the two significant linkages, 5 additional genes of the screening set demonstrated statistically suggestive linkage to proximal mouse chromosome 17 (*Serping1, Col1a2, Nid1, Mmp2, Mfap5*). The confidence intervals for the suggestive peaks also were in general somewhat broader than the genes with significant linkage, but all encompassed the 8-33Mb interval shared by the *Col1a1* and *Fbn1* eQTLs (Table 1, above).

These results suggest the presence of an ECM-associated eQTL that controls a significant number of ECM genes. To test this possibility, the genes whose gene expression where most highly associated with the inheritance of a particular SNP was determined for the proximal portion of chromosome 17. The trait correlation function of WebQTL was used to identify the top 100 probe sets associated with inheritance of either the DBA/2J or AKR/J allele. Gene ontology (GO) analysis was subsequently performed to determine whether ECM-associated genes were over-represented, as would be predicted by the presence of an ECM master transcriptional regulator. Analysis of SNPs spaced at approximately 7Mb intervals from 0 to ~60 megabase of the chromosome revealed a significant over-representation of ECM-associated genes for 6 SNPs, with as many as 16 of the top 100 correlated genes identified as ECM components (*P*=4x10^-13^) (**Table 2**, below). eQTL analysis of the new ECM-associated genes identified 5 additional statistically significant ECM eQTL loci (*Col6a2, Cilp, Col5a2, Postn, Col8a1)*. Taken together, these data support the presence of at least one common ECM eQTL, most likely located in the ~7-33 megabase interval on the chromosome.

**Table 2: Results of trait correlation gene ontology analysis**

| Locus | Physical location (Mb^*^) | Number of ECM genes^§^ | *P* value^†^ |
| --- | --- | --- | --- |
| Rs6310831 | 3.2 | 4 | 1.4 x 10^-4^ |
| Rs3090641 | 7.7 | 16 | 4.0 x 10^-13^ |
| Rs6181074 | 14.3 | 16 | 4.0 x 10^-13^ |
| Rs3032864 | 22.0 | 16 | 4.0 x 10^-13^ |
| Rs13482947 | 29.4 | 15 | 3.0 x 10^-12^ |
| Rs3682923 | 33.6 | 8 | 1.5 x 10^-5^ |
| Rs6409750 | 44.9 | 0 | NA^‡^ |
| Rs13483042 | 53.1 | 0 | NA |
| mCV22888090 | 61.3 | 0 | NA |

*Megabase position from centromere

^§^Number of genes in top 100 correlated identified as extracellular matrix components by Gene Onotology analysis

^†^Significance for enrichment of ECM genes in trait correlation gene ontology analysis

^‡^Not applicable

**Tumor progression and metastasis QTL analysis**

Analysis of the tumor growth and progression locus on proximal chromosome 17 is described in Lancaster et al[5]. Briefly, a weak potential metastasis QTL was observed in the original multicross analysis used for initial mapping of *Mtes1*[6]. To further investigate this observation, chromosomal substitution strains were generated and characterized[5]. Significant suppression of tumor growth and metastatic progression was observed, confirming the presence of one or more tumor progression modifiers somewhere on chromosome 17. To attempt to achieve subchromosomal mapping resolution of the modifier gene(s), composite interval mapping was performed on the original experimental backcrosses. A significant tumor progression locus was discovered on proximal chromosome 17 in the I/LnJ backcross when controlled for the previously identified *Mtmg* loci on chromosomes 4 and 7. The peak linkage of the new tumor progression locus was ~30Mb from the centromere, with the 95% confidence interval spanning the interval from ~0-40 megabase from the centromere. To gain a better approximation of the most likely position of the modifier gene bootstrap resampling of the data was performed. This analysis assigned the most likely location of the tumor progression modifier in the proximal 30 megabase of the chromosome 79% of the time, the same interval observed for the most likely location of the ECM eQTL (see figure 1 of Lancaster et al[5]).

**Conclusions**

The most likely locations of the ECM eQTL and tumor progression modifier co-localize, which is entirely consistent with the hypothesis that they may be due to shared causative polymorphisms. Within the limits of the resolution achievable by the available analysis, it is not possible to further resolve or refine the map location of the QTLs. The interval in question, however, contains a large number of genes, and the genetic analysis by itself is insufficient to validate this hypothesis. Identification of candidate genes therefore necessitates additional *in vitro* and *in vivo* analysis to further investigate the origins of the observed linkage.

*Anakin* is one of thirty genes located within a genomic region spanning the peak eQTL linkage region that displays both a high degree of correlation and a low *P* value with regard to expression of two or more of the 9 probes within metastasis-predictive ECM genes (**Supplementary Table 3**). However, *Anakin* was deemed a priority for further analysis for the following reasons: a) its physical proximity to the peak eQTL linkage; b) its apparent correlation with expression of various metastasis-predictive ECM genes; and c) that it had also been identified as interacting with the metastasis efficiency modifier *Sipa1*. The effects upon metastasis of other genes within the eQTL linkage region remain under investigation, but their potential role in the modulation of metastasis efficiency (if any) is beyond the scope of the current study.

References

1. Yang H, Crawford N, Lukes L et al (2005) Metastasis Predictive Signature Profiles Pre-exist in Normal Tissues. Clin Exp Metastasis 22:593-603

2. Wang J, Williams RW, Manly KF (2003) WebQTL: web-based complex trait analysis. Neuroinformatics 1:299-308

3. Chesler EJ, Lu L, Shou S et al (2005) Complex trait analysis of gene expression uncovers polygenic and pleiotropic networks that modulate nervous system function. Nat Genet 37:233-242

4. Ramaswamy S, Ross KN, Lander ES et al (2003) A molecular signature of metastasis in primary solid tumors. Nat Genet 33:49-54

5. Lancaster M, Rouse J, Hunter KW (2005) Modifiers of mammary tumor progression and metastasis on mouse chromosomes 7, 9, and 17. Mamm Genome 16:120-126

6. Hunter KW, Broman KW, Voyer TL et al (2001) Predisposition to efficient mammary tumor metastatic progression is linked to the breast cancer metastasis suppressor gene Brms1. Cancer Res 61:8866-8872
