# Supplementary material for: Rrp1b, a New Candidate Susceptibility Gene for Breast Cancer Progression and Metastasis
Source: PLoS Genet. 2007 Nov 30;3(11):e214. doi: 10.1371/journal.pgen.0030214 (PMC2098807; doi:10.1371/journal.pgen.0030214)
Supplement: Text S2 — (62 KB PDF) [file pgen.0030214.sd002.pdf]

Yeast Two Hybrid Method at Myriad

Myriad Genetics has developed an automated process for the large-scale identification of protein-protein interactions that is based on the nuclear yeast two-hybrid methodology originally developed in the late 80ies by the Fields laboratory (Fields and Song, Nature 340:245-246, 1989.). This technology has been reviewed in the book "The Yeast Two Hybrid System" (eds. Bartel and Fields, New York: Oxford University Press, Inc., 1997) and several articles (Miller and Stagljar, Methods Mol Biol. 261:247-262, 2004; Gietz and Woods, Methods Mol Biol. 185:471-486, 2002; Toby and Golemis, Methods 24:201-217, 2001; Bai and Elledge, Methods Enzymol 273:331-347, 1996; Fields and Sternglanz, Trends Genet 10:286-292, 1994). Dr. Paul Bartel, who trained in the laboratory of Dr. Stanley Fields, started in 1997 with the assembly of a team of Myriad scientists and programmers to adapt the system for large-scale application. This involved the development of novel reagents, such as strains and plasmid vectors, and techniques. Additionally, the application of Myriad's advanced robotics and bioinformatics capabilities further developed the technology into a robust and high fidelity platform that has been termed ProNet (for Protein Network). Myriad has the following technological advantages that allow to reliably discover large numbers of protein-protein interactions:

- A roboticized bait creation process that allows for the construction of multiple 48 DNA-binding domain constructs to be made in parallel.
- A process for constructing high complexity custom activation domain libraries that are made directly in yeast by means of homologous recombination.
- An efficient yeast mating strategy that allows thorough screening of activation domain libraries.
- Automation and tracking of all elements in the process through a sophisticated LIMS database, providing a means of ensuring high accuracy and throughput.
- Software for the management of the process and analysis of protein interaction networks.

Below is a brief description of the ProNet technology.

Media and Reagents:
The drop out media and plates are prepared according to Guthrie and Fink (Guide to Yeast Genetics and Molecular Biology, Methods in Enzymology, Vol 194, 1991, Academic Press, Inc). PCR reagents are purchased from Sigma-Aldrich Co. (St. Louis, MO), Applied Biosystems (Foster City, CA), and Stratagene (La Jolla, CA), and are prepared according to recipes developed at Myriad Genetics. ProNet uses robust quality control procedures to ensure the quality of all the reagents and media.

Plasmids:

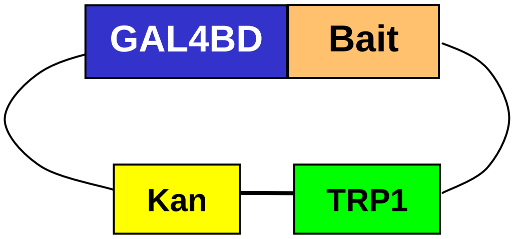

The current DNA-binding domain vector for constructing baits in ProNet is pGBT.superB. It carries an *E. coli* origin of replication (ori) and a kanamycin resistance gene (Kan) for maintenance and selection of the plasmid in *E. coli*. The *S. cerevisiae* TRP1 gene is included for selection in yeast, and CEN and ARS sequences are included for maintenance of the plasmid at single copy in yeast. Baits are placed in-frame, C-terminal to the Gal4 DNA-binding domain (nucleotide 1-441 coding sequence in GAL4), followed by the ADH1 transcriptional terminator sequence. The ADH1 promoter drives expression of the fusion between the Gal4 DNA-binding domain and bait. This vector also contains a 1.6 kb CEN6 sequence inserted into the multiple cloning site between EcoR I and Sal I sites. This prevents empty bait vector from propagating in yeast.

The ProNet activation domain vector for construction of prey libraries is pGAD.PN2. It carries an *E. coli* origin of replication (ori) and an ampicillin resistance gene (Amp) for maintenance and selection of the plasmid in *E. coli*. The *S. cerevisiae* LEU2 gene is included for selection in yeast, and CEN and ARS sequences are included for

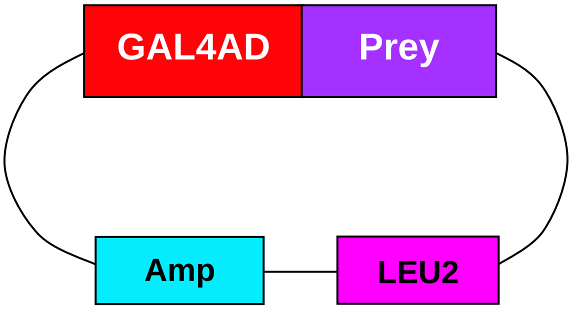

maintenance of the plasmid at single copy in yeast. Preys are placed C-terminal to the Gal4 activation domain (nucleotide 2301-2643 coding sequence in GAL4), followed by the PGK1 transcriptional terminator sequence. The ADH1 promoter drives expression of the fusion between the Gal4 activation domain and prey.

Yeast Strains:
The ProNet yeast strain used to maintain the bait plasmids is PNY200 (*MATα ura3-52 ade2-101 trp1-901 his3-Δ200 leu2-3,112 gal4Δ gal80Δ*). The ProNet yeast strain used to maintain the prey constructs is BK100 (*MAT***a** *ura3-52 trp1-901 his3-Δ200 leu2-3,112 gal4Δ gal80Δ GAL2-ADE2 LYS2::GAL1-HIS3 met2::GAL7-lacZ*). This strain is a derivative of PJ69-4A (James et al., Genetics 144:1425-1436, 1996).

Prey library construction (ADL: activation domain library)
ProNet uses double polyA selected mRNA for ADL construction. First strand cDNA synthesis is initiated by priming with random decamers that contain a common tag sequence and a biotin blocker. Second strand synthesis is performed according to the Gubler-Hoffman procedure, followed by blunt-ending with T4 DNA polymerase. The cDNA is purified and ligated with a DNA adaptor. The adaptor-ligated cDNA is subjected to gel filtration to remove free adaptors, followed by PCR amplification with a pair of primers that anneal to the 5’ and 3’ tags and contain tails for homologous recombination in yeast. The PCR-amplified DNA is gel purified to remove fragments containing inserts shorter than ~250 bp and co-transformed with linear pGAD.PN2 vector into BK100 for homologous recombination in vivo. The yeast transformants are harvested from the selection plates and dispensed into aliquots for –80 °C storage for future library screening experiments. The majority of ProNet ADLs consist of more than 10 million primary transformants.

Bait construction
ProNet uses a proprietary primer design program to select optimal primer pairs for bait construction. The primers contain tails for secondary amplification and recombination into the bait vector pGBT.superB. Primer pairs are ordered in 96-well plates. After a primary PCR, the products are gel purified and subjected to a proprietary, secondary round of PCR that generates a bait fragment with tails long enough for homologous recombination in yeast. The bait fragment with tails is then co-transformed with linear pGBT.superB DNA (excluding the CEN6

fragment) into PNY200 to obtain recombinant plasmid in vivo. Eight individual yeast colonies are picked for each bait strain and subjected to size verification by PCR/gel electrophoresis and sequence confirmation.

Yeast two hybrid searches and identification of bait-prey interactions
ProNet uses a mating based method to screen for bait-prey interactions. Approximately 25-30 million MATα yeast cells containing single bait are mixed with 60 million MAT**a** library yeast cells and allowed to mate on filters. Between 5-10 million diploid yeast cells are routinely obtained for each mating. After mating the cells are plated onto selective media. Transcription of two auxotrophic reporter genes (HIS3 and ADE2) with dissimilar promoters (see BK100 genotype) occurs if the bait and prey protein interact. Colonies are picked from the selection plates, and the prey inserts are identified by sequence analysis.

To confirm the interactions, the bait and prey plasmids are isolated from yeast diploids and electroporated into *E. coli*. Both bait and prey plasmids are purified followed by sequencing to confirm their identities. The bait and prey plasmid DNAs are co-transformed into a naïve yeast strain to recapitulate the interaction. The confirmation test takes advantage of a third reporter gene (lacZ) and is based on a chemiluminescent reporter gene assay system. The specificity of the prey is investigated in a separate false positive test where the prey is tested against a mixture of several heterologous baits.

Final analysis and interaction release
Bait and prey sequencing information with their associated BLAST results, as well as the results from bench confirmation assays are deposited into a database. The sequences are subjected to a proprietary automatic analysis program, and the results are displayed in a web browser for scientists' review and release of interactors.
